# A super pan‐genome map provides genomic insights into evolution of diploid cotton species

**DOI:** 10.1002/imo2.15

**Published:** 2024-06-27

**Authors:** Xueqiang Wang, Hejun Lu, Yan Zhao, Zhiyuan Zhang, Jun Li, Zeyu Dong, Yupeng Hao, Lei Fang, Xueying Guan, Ting Zhao, Yan Hu, Tianzhen Zhang

**Affiliations:** ^1^ Zhejiang Provincial Key Laboratory of Crop Genetic Resources The Advanced Seed Institute, Plant Precision Breeding Academy, College of Agriculture and Biotechnology Zhejiang University Hangzhou Zhejiang China; ^2^ Cotton and Economic Crops Precision Breeding & Germplasm Innovation Team Hainan Institute of Zhejiang University Sanya Hainan China; ^3^ Seed Production and Quality Control Research Center Hainan Yazhou Bay Seed Laboratory Sanya Hainan China; ^4^ State Key Laboratory of Crop Biology, Shandong Key Laboratory of Crop Biology College of Agronomy Shandong Agricultural University Tai'an Shandong China

## Abstract

A high‐quality super pan‐genome was built using 22 representative diploid cottons species. Adaptive evolution among extant Gossypium species was investigated. Specific genes were enriched for different terms, revealing variations in characteristics of different cotton species. The 321 hotspot regions of structural variations (SVs), containing 90 genes associated with fiber initiation and/or elongation, were detected. A 444‐bp deletion in the promoter sequence of *GoNe* that explained the lack of foliar nectary in *G. gossypiodes* (D6) and *G. schwendimanii* (D11) was identified.

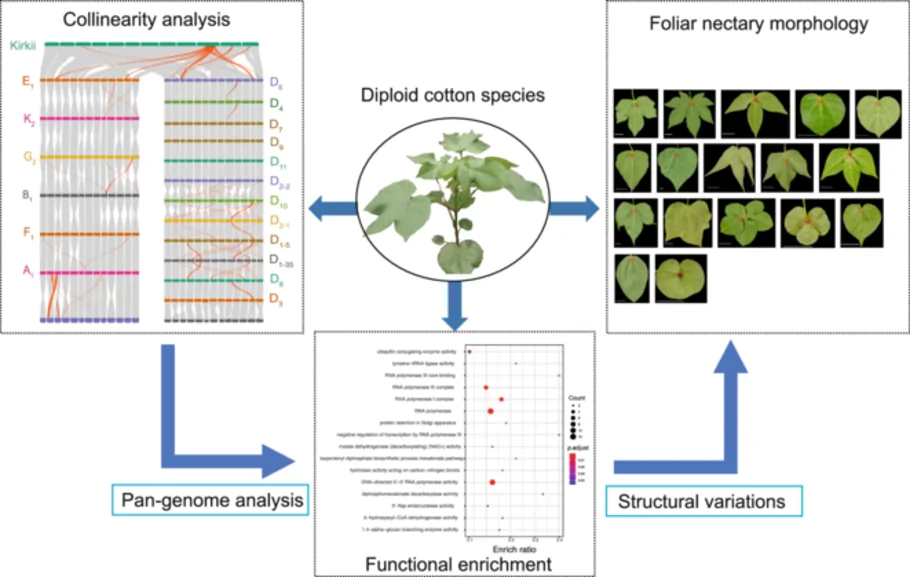

To the editor,

The *Gossypium* genus can be divided into eight diploid cotton groups (A, B, C, D, E, F, G, and K genomes), comprising 45 diploid species. Inferring ancestral genomes (IAG) among extant *Gossypium* species is an important goal of comparative genomics, and several mathematical models and approaches have been proposed for IAG. Recently, a new framework, inferring ancestor genome structure (IAGS), was described [1]. Over the last decades, there have been several efforts to deeply sequence many diploid cotton species, but pan‐genomic analyses have mainly focused on tetraploid cotton species [2, 3, 4, 5, 6, 7]. A tetraploid cotton pan‐genome was constructed by combining newly sequenced genomes of *Gossypium hirsutum* L. and two other wild species with sequences from five previously published tetraploid cotton species [8]. A pan‐genome including 10 representative *Gossypium* diploid genomes linked changes in chromatin structures to phenotypic differences in cotton fiber and identified regulatory variations that control the genetic basis of fiber length [2]. This study focused on one D_5_ genome, with less attention to other species of the d‐genome. The recent article mainly delves into the evolutionary history and the mechanisms underlying the rapid adaptive radiation of *Gossypium*, with a particular focus on the roles of incomplete lineage sorting and gene flow [9]. Thus, the purpose of our study was to construct a pan‐genome and carry out in‐depth comparative genome analysis for diploid cotton including all species of d‐genome and the other species of *Gossypium*, and perform the comparative genomic analysis for nectary development gene (*GoNe*) in different species of *Gossypium* [10].

In this study, we investigated 22 diploid cotton species and their wild relative *Gossypioides kirkii* (Mast.) J. B. Hutch. [11] with high‐quality genomes publicly available in the CottonGen (https://www.cottongen.org/), IAGS among extant *Gossypium* species, and constructed a super pan‐genome of cultivated and wild diploid cotton species. The structural variations (SVs) in different diploid cotton were examined and hotspot regions of SVs were detected. We investigated the presence or absence of foliar nectary in 17 diploid cotton species and carried out the comparative genomic analysis of *GoNe* in different species [10].

The 22 diploid cotton genomes were used for pan‐genomic analysis, represented by seven of the eight recognized genome groups and 19 representative diploid cotton species, including its wild relative *G. kirkii* (Kirkii). The assembly completeness of each genome was evaluated with Benchmarking Universal Single‐Copy Orthologs evaluation (Table S1). Transposable elements (TEs) were annotated to classify and assess their distribution in the cotton genomes. The most TEs were found in *Gossypium rotundifolium* Fryxell et al. (K_2_), and the fewest in wild relative Kirkii (Figure 1A; Figure S1; Table S2). There was a significant increase in the length of TEs from the D genome to the G and K genomes, and from the B, E, and F genomes to the A genome, suggesting that TEs may have exerted important influences on the evolution of cotton. There was a significant positive correlation between the proportions of TEs in total sequence per genome and assembly length, suggesting that the increase of TEs might contribute to genome amplification (Figure 1A; Figure S1; Table S2). Gypsy and Copia long terminal repeats were identified as significant contributors to the genome amplification process.

**Figure 1:**
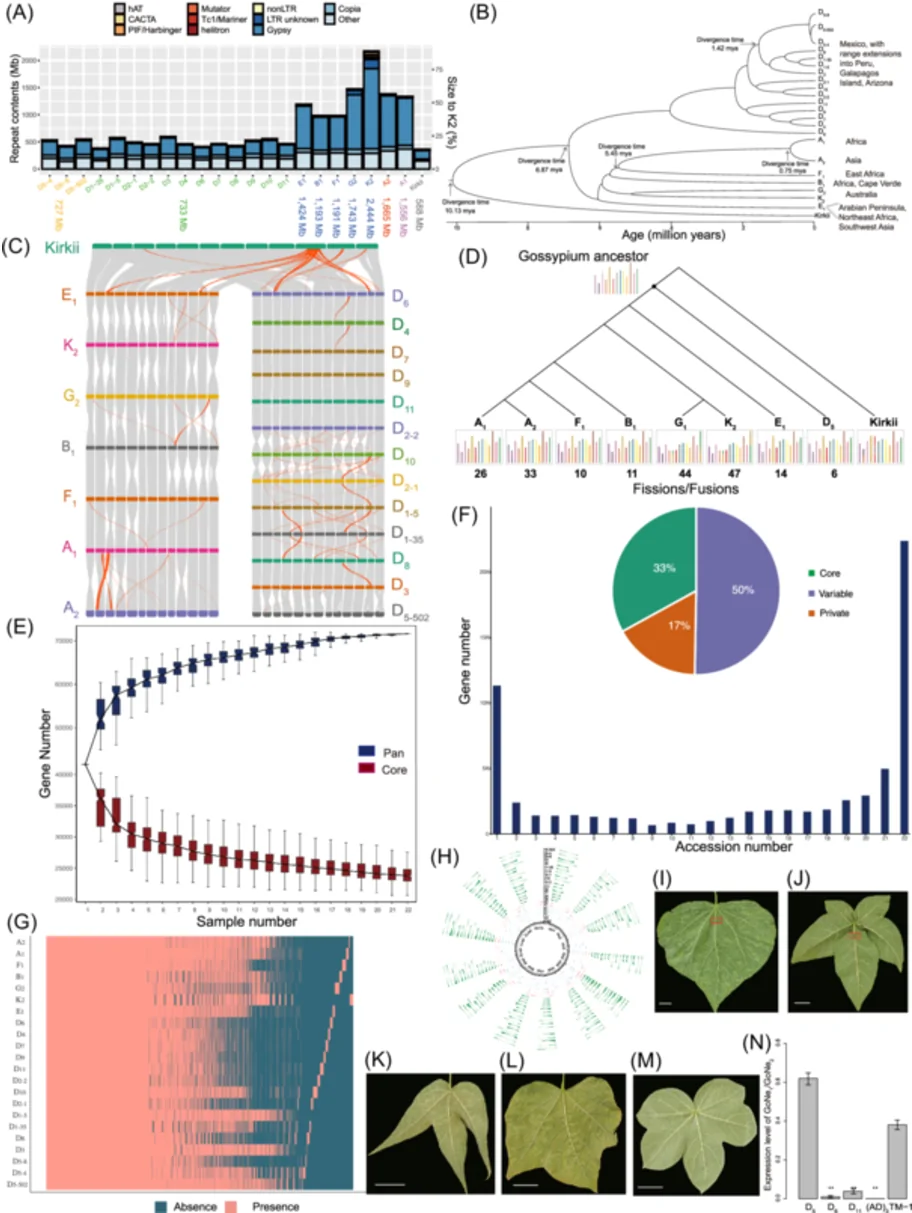
Exploring the genomic diversity and adaptive evolution of diploid cotton species through pan‐genomic analysis. (A) The transposable element length and assembly length for the different cotton species. The different colors of genomes represented different diploid cotton genomes. (B) Phylogenetic tree and divergence times of 27 genomes using 352 single‐copy coding genes with the phylogenetic outgroup species *Kirkii*. Arrows on the graph represented the divergence times of different genomes. (C) Collinearity blocks between two genomes based on the phylogenetic tree. The different colors in chromosomes represented different cotton genomes. The gray links between the two genomes represented collinear blocks, while the orange links between the two genomes represented disordered collinear blocks. (D) Ancestor genome construction and synteny block fission and fusions. (E) Variation of genes in the pan‐genome and core genome along with an additional diploid cotton genome. (F) Compositions of the pan‐genome and individual genomes. The histogram shows the number of genes in the 22 genomes with different frequencies. (G) Clustering of core and dispensable genes of diploid cotton genomes. (H) The distributions of structural variations (SVs) in different diploid cotton genomes. The colored histograms in each layer from inner to outer represented, respectively, the SVs detected from each diploid cotton genome. Kirkii: *Gossypioides kirkii*, G_2_: *Gossypium austral*, F_1_: *Gossypium longicalyx*, E_1_: *Gossypium stocksii*, B_1_: *Gossypium anomalum*, A_1_: *Gossypium herbaceum*, A_2_: *Gossypium arboreum*, D_6_: *Gossypium gossypioides*, D_4_: *Gossypium aridum*, D_7_: *Gossypium lobatum*, D_9_: *Gossypium laxum*, D_11_: *Gossypium schwendimanii*, D_2‐2_: *Gossypium harknessii*, D_10_: *Gossypium turneri*, D_2‐1_: *Gossypium armourianum*, D_1‐5_: *Gossypium thurberi*, D_1‐35_: *Gossypium thurberi*, D_8_: *Gossypium trilobum*, D_3_: *Gossypium davidsonii*, D_5‐8_: *Gossypium raimondii*, D_5‐4_: *G. raimondii*, D_5‐502_: *G. raimondii*. Purple represented the outgroup, light blue represented other cotton groups (B, E, F, and G genomes), red represented the cotton group (A genome), and green represented the cotton group (D genome). (I–N) Investigation of foliar nectary and expression level of *GoNe*_1_ */GoNe*_2_ in five diverse cotton species, (I) D_5_; (J) TM‐1 (AD)_1_; (K) D_6_; (L) D_11_; (M) (AD)_3_; (N) Expression of *GoNe*_1_ */GoNe*_2_ in five diverse cotton species. The red frame is foliar nectary in each species. (AD)_3_, D_6_, and D_11_ did not have any nectaries on the midribs of leaves. The scale bar represented 2 cm. **Significant differences (*p* < 0.05) detected by one‐way analysis of variance between the presence of foliar nectaries (TM‐1 and D_5_) to the absence of foliar nectaries [(AD)_3_, D_6_ and D_11_].

The genetic relationships, evolution, and divergence time of 22 diploid cotton genomes were then analyzed using whole‐genome sequencing. A maximum‐likelihood phylogenetic tree was constructed using 352 single‐copy coding genes, revealing two distinct clades with D genome diploid cotton species forming one clade. The cultivated diploid cotton diverged from the wild diploid cotton species about 5.45 Mya, and the divergence time between diploid cotton species and its wild relative *G. kirkii* was about 10.13 Mya (Figure 1B; Figure S2). Collinearity blocks between assembly genomes were identified, and 13 collinearity blocks between *Gossypium herbaceum* L. (A_1_) and *Gossypium arboreum* L. (A_2_) were disordered on different corresponding genomes, suggesting their importance in diploid cotton evolution (Figure 1C). The *Gossypium* ancestor genome was inferred using the genome median problem model, and chromosome fission and inversions were found to be fundamental forces for speciation. Huge chromosome inversions may drive species formation and diversity (Figure 1D; Figure S3). Future studies will investigate the causes of fission and whether it was caused by selection or upheaval environment.

We then constructed a super pan‐genome through evaluation of 22 genomes and gene annotations of diploid cotton species. The pan‐genome contained 67,807 genes, including 22,384 core, 34,093 variable, and 11,330 specific genes (Figure 1E–G; Tables S3–5). Kyoto Encyclopedia of Genes and Genomes pathway and gene ontology enrichment analysis of core genes showed terms related to growth and development of cotton (Table S6). Specific genes were enriched for different terms, revealing variations in characteristics of different cotton species [2, 3, 4] (Figures S4–12; Tables S7–13). For instance, specific genes in *G. herbaceum* (A_1_) and *G. arboreum* (A_2_) were related to disease resistance and lint yield, respectively (Figures S4 and S5; Tables S8 and S9). Specific genes in *Gossypium raimondii* Ulbr. (D_5_) were associated with biomass, fiber quality, and stress/disease resistance (Figures S6 and S7; Table S10). The specific genes in *Gossypium anomalum* Waw. & Peyr. (B_1_) were linked to drought tolerance (Figure S11; Table S12) [3], corresponding to their species‐specific characteristics.

To overcome reference genome bias, SVs were identified in 22 diploid cotton assembly genomes using three reference genomes. Results showed differences in total number and types of SVs across cotton species (Figure 1H; Figures S13–16; Tables S14–17). Repeat contraction was most common and deletion least common. *Gossypium armourianum* Kearney (D_2‐1_) had the greatest number of SVs and *G. kirkii* had the fewest. Wild D genomes had a 1.5‐fold greater number of SVs than cultivated cotton species (A_1_ and A_2_ genomes), and there were no significant differences in SVs between wild (B_1_, E_1_, F_1_, and G_2_ genomes) and cultivated cotton species (A_1_ and A_2_ genomes) using K_2_ as reference (Figures S13 and S16; Table S14). Unevenly distributed SVs were identified in 321 SV hotspot regions, including 90 genes associated with fiber initiation and/or elongation (Figure S16; Table S18). A_2_ genome had fewer SVs in these regions, possibly explaining its high lint yield and fiber quality. This could be attributed to the fact that the genes associated with fiber initiation and/or elongation in the A_2_ genome were less impacted by SVs.

Finally, foliar nectaries in *Gossypium* provide the plant with defense against herbivores [10], and phenotypic investigations were conducted on 17 cotton species, classified into seven diploid cotton groups, to determine the presence of foliar nectaries. No foliar nectary was found in *Gossypium gossypiodes* (Ulbr.) Standl. (D_6_), consistent with previous studies [12], *Gossypium schwendimanii* Fryxell & S. D. Koch (D_11_), and *Gossypium tomentosum* Nutt. ex Seem. ((AD)_3_), the allotetraploid cotton species (Figure 1I,M; Figure S17). Comparative genomic analysis of *GoNe* expression revealed no expression in D_6_ and D_11_, suggesting a lack of function of *GoNe* in preventing foliar nectary development in these two wild diploid species (Figure 1N; Figure S18; Table S19). Sequence analysis of the *GoNe* promoter sequences showed a large deletion (444‐bp fragment) in the promoter sequences of *GoNe* from D_6_ and D_11_ species compared to other diploid cotton species with foliar nectaries (Figures S19 and S20).

In conclusion, we constructed a high‐quality super pan‐genome of 22 diploid cotton species and investigated their adaptive evolution. The pan‐genome contained 67,807 genes, including core, variable, and specific genes, and identified SVs and hotspot regions associated with fiber initiation and/or elongation. The study also investigated the absence of foliar nectary in *G. gossypiodes* and *G. schwendimanii*, and identified the deletion in the promoter sequence of *GoNe* as the cause. This study provides insights into the genetic diversity of diploid cotton and its dynamic genomic variation during expansion, which can aid modern cotton breeding.

## AUTHOR CONTRIBUTIONS

**Xueqiang Wang**: Conceptualization; methodology; investigation; formal analysis; funding acquisition; writing—original draft. **Hejun Lu**: Formal analysis; conceptualization; methodology; writing—review and editing. **Yan Zhao**: Conceptualization; methodology; formal analysis; writing—review and editing; funding acquisition. **Zhiyuan Zhang**: Formal analysis; conceptualization; methodology; writing—review and editing; funding acquisition. **Jun Li**: Conceptualization; writing—review and editing. **Zeyu Dong**: Investigation. **Yupeng Hao**: Investigation. **Lei Fang**: Conceptualization; writing—review and editing. **Xueying Guan**: Conceptualization; writing—review and editing. **Ting Zhao**: Investigation; writing—review and editing. **Yan Hu**: Conceptualization; writing—review and editing; supervision. **Tianzhen Zhang**: Conceptualization; supervision; writing—review and editing; funding acquisition.

## CONFLICT OF INTEREST STATEMENT

The authors declare no conflict of interest.

## ETHICS STATEMENT

No animals or humans were involved in this study.

## Supporting information


**Figure S1:** The types and numbers of transposable elements (TEs) and TE length/assembly length in different cotton species. The different colors of genomes represented different diploid cotton.
**Figure S2:** Phylogenetic tree of twenty‐two genomes using 352 single‐copy coding genes with the phylogenetic outgroup species *Gossypioides kirkii* (Kirkii).
**Figure S3:** Ancestor genome construction and dotplot based on the synteny blocks.
**Figure S4:** Pathway (A) and Gene ontology (GO) (B) enrichment of specific genes unique in A_1_ genome.
**Figure S5:** Pathway (A) and Gene ontology (GO) (B) enrichment of specific genes unique in A_2_ genomes.
**Figure S6:** Pathway and gene ontology (GO) enrichment of specific genes unique in D_5‐502_ genome.
**Figure S7:** Gene ontology (GO) enrichment of specific genes unique in D_5‐4_ (A) and D_5‐8_ (B) genomes.
**Figure S8:** Gene ontology (GO) enrichment of specific genes unique in D_1‐5_ (A) and D_8_ (B) genomes.
**Figure S9:** Gene ontology (GO) enrichment of specific genes unique in D_3_ (A) and D_10_ (B) genomes.
**Figure S10:** Gene ontology (GO) enrichment of specific genes unique in B_1_ (A), E_1_ (B) and G_2_ (C) genomes.
**Figure S11:** The biological process (A), cellular component (B) and molecular function (C) in gene ontology (GO) enrichment of specific genes unique in K_2_ genome.
**Figure S12:** Pathway enrichment of specific genes unique in K_2_ genome.
**Figure S13:** The types and numbers of SVs in different diploid cotton using the K_2_ reference.
**Figure S14:** The types and numbers of SVs in different diploid cotton using the D_5‐502_ reference.
**Figure S15:** The types and numbers of SVs in different diploid cotton using the A_2_ reference.
**Figure S16:** The distributions of SVs in different diploid cotton genomes.
**Figure S17:** Investigation of foliar nectary in 17 diverse cotton species.
**Figure S18:** The expression level of *GoNe*_1_ (A) and *GoNe*_2_ (B) in five diverse cotton species.
**Figure S19:** Sequence differences of CDS from *GoNe*_1_ (for A subgroup) and *GoNe*_2_ (for D subgroup) in the diploid cotton species.
**Figure S20:** Sequence differences of promoter from *GoNe*_1_ (for A subgroup) and *GoNe*_2_ (for D subgroup) in the diploid cotton species.

**Table S1:** Information and assessment of twenty‐three genomes.
**Table S2:** The masked sequence and numbers of transposable elements and TE length/assembly length in different cotton species.
**Table S3:** The IDs and information of genes in our pan‐genome.
**Table S4:** The gene PAVs in different diploid cottons.
**Table S5:** The gene number in genome numbers of different diploid cotton.
**Table S6:** Pathway and gene ontology (GO) enrichment of core genes.
**Table S7:** The specific gene number to each assembly of diploid cotton.
**Table S8:** Pathway and gene ontology (GO) enrichment of specific genes unique in A_1_ genome.
**Table S9:** Pathway and gene ontology (GO) enrichment of specific genes unique in A_2_ genomes.
**Table S10:** Pathway and gene ontology (GO) enrichment of specific genes unique in D_5_ genomes.
**Table S11:** Pathway and gene ontology (GO) enrichment of specific genes unique in D genomes except D_5_.
**Table S12:** Pathway and gene ontology (GO) enrichment of specific genes unique in other genomes except A and D genomes.
**Table S13:** Pathway and gene ontology (GO) enrichment of specific genes unique in Kirkii genome.
**Table S14:** The size range and number of SVs in different diploid cotton using the K_2_ reference.
**Table S15:** The size range and number of SVs in different diploid cotton using the D_5‐502_ reference.
**Table S16:** The size range and number of SVs in different diploid cotton using the A_2_ reference.
**Table S17:** Location of the detected SVs on the genome of 22 cotton species.
**Table S18:** List of 321 SV hotspot regions and 90 genes associated with fiber initiation or/and elongation.
**Table S19:** Oligonucleotides used for qRT‐PCR in this study.

## Data Availability

The assembled genomes of 27 diploid cotton and one phylogenetic outgroup species *Gossypioides kirkii* (Kirkii) were downloaded from the CottonGen (https://www.cottongen.org/) and the detailed information (DOI and URL) is included in Table S1. The plot code has been submitted to Github (https://github.com/xqwang1990/Cotton_Pangenome_Plot). Supporting Information (methods, figures, tables, scripts, graphical abstract, slides, videos, Chinese translated version, and update materials) may be found in the online DOI or iMeta Science https://www.imeta.science/imetaomics/.
