# Supplementary material for: A super pan‐genome map provides genomic insights into evolution of diploid cotton species
Source: IMetaOmics. 2024 Jun 27;1(1):e15. doi: 10.1002/imo2.15 (PMC12806262; doi:10.1002/imo2.15)
Supplement: Supplementary file 1 — Figure S1: The types and numbers of transposable elements (TEs) and TE length/assembly length in different cotton species. The different colors of genomes represented different diploid cotton. Figure S2: Phylogenetic tree of twenty‐two genomes using 352 single‐copy coding genes with the phylogenetic outgroup species Gossypioides kirkii (Kirkii). Figure S3: Ancestor genome construction and dotplot based on the synteny blocks. Figure S4: Pathway (A) and Gene ontology (GO) (B) enrichment of specific genes unique in A1 genome. Figure S5: Pathway (A) and Gene ontology (GO) (B) enrichment of specific genes unique in A2 genomes. Figure S6: Pathway and gene ontology (GO) enrichment of specific genes unique in D5‐502 genome. Figure S7: Gene ontology (GO) enrichment of specific genes unique in D5‐4 (A) and D5‐8 (B) genomes. Figure S8: Gene ontology (GO) enrichment of specific genes unique in D1‐5 (A) and D8 (B) genomes. Figure S9: Gene ontology (GO) enrichment of specific genes unique in D3 (A) and D10 (B) genomes. Figure S10: Gene ontology (GO) enrichment of specific genes unique in B1 (A), E1 (B) and G2 (C) genomes. Figure S11: The biological process (A), cellular component (B) and molecular function (C) in gene ontology (GO) enrichment of specific genes unique in K2 genome. Figure S12: Pathway enrichment of specific genes unique in K2 genome. Figure S13: The types and numbers of SVs in different diploid cotton using the K2 reference. Figure S14: The types and numbers of SVs in different diploid cotton using the D5‐502 reference. Figure S15: The types and numbers of SVs in different diploid cotton using the A2 reference. Figure S16: The distributions of SVs in different diploid cotton genomes. Figure S17: Investigation of foliar nectary in 17 diverse cotton species. Figure S18: The expression level of GoNe 1 (A) and GoNe 2 (B) in five diverse cotton species. Figure S19: Sequence differences of CDS from GoNe 1 (for A subgroup) and GoNe 2 (for D subgroup) in the diploid cotto [file IMO2-1-e15-s001.docx]

**Supplementary Information**

**Title:** A super pan-genome map provides genomic insights into evolution of diploid cotton species

Running Title: Pan-genomic landscape of diploid cottons

Xueqiang Wang^1,2,3#^, Hejun Lu^1#^, Yan Zhao^4#^, Zhiyuan Zhang^2#^, Jun Li^1,2,3^, Zeyu Dong^1,2^, Yupeng Hao^1,2^, Lei Fang^1^, Xueying Guan^1^, Ting Zhao^1^, Yan Hu^1^ and Tianzhen Zhang^1*^

^1^ Zhejiang Provincial Key Laboratory of Crop Genetic Resources, the Advanced Seed Institute, Plant Precision Breeding Academy, College of Agriculture and Biotechnology, Zhejiang University, Hangzhou, 310058, China.

^2^ Hainan Institute of Zhejiang University, Sanya, Hainan, 572025, China.

^3^ Hainan Yazhou Bay Seed Laboratory, Sanya, Hainan, 572025, China.

^4^ State Key Laboratory of Crop Biology, Shandong Key Laboratory of Crop Biology, College of Agronomy, Shandong Agricultural University, Tai'an, Shandong, 271018, China.

^#^ These authors have contributed equally to this work.

^*^ Correspondence should be addressed to Tianzhen Zhang (Email: cotton@zju.edu.cn).

**This PDF file includes:**

Supplemental Methods

Figures S1-S20

Tables S1-S19

Supplemental References

**LIST OF SUPPLEMENTAL INFORMATION**

**Supplemental Methods**

**Supplemental Figures**

**Figure S1** The types and numbers of transposable elements (TEs) and TE length/assembly length in different cotton species. The different colors of genomes represented different diploid cotton.

**Figure S2** Phylogenetic tree of twenty-two genomes using 352 single-copy coding genes with the phylogenetic outgroup species *Gossypioides kirkii* (Kirkii).

**Figure S3** Ancestor genome construction and dotplot based on the synteny blocks.

**Figure S4** Pathway **(A)** and Gene ontology (GO) **(B)** enrichment of specific genes unique in A_1_ genome.

**Figure S5** Pathway **(A)** and Gene ontology (GO) **(B)** enrichment of specific genes unique in A_2_ genomes.

**Figure S6** Pathway and gene ontology (GO) enrichment of specific genes unique in D_5-502_ genome.

**Figure S7** Gene ontology (GO) enrichment of specific genes unique in D_5-4_ **(A)** and D_5-8_ **(B)** genomes.

**Figure S8** Gene ontology (GO) enrichment of specific genes unique in D_1-5_ **(A)** and D_8_ **(B)** genomes.

**Figure S9** Gene ontology (GO) enrichment of specific genes unique in D_3_ **(A)** and D_10_ **(B)** genomes.

**Figure S10** Gene ontology (GO) enrichment of specific genes unique in B_1_ **(A)**, E_1_ **(B)** and G_2_ **(C)** genomes.

**Figure S11** The biological process **(A)**, cellular component **(B)** and molecular function **(C)** in gene ontology (GO) enrichment of specific genes unique in K_2_ genome.

**Figure S12** Pathway enrichment of specific genes unique in K_2_ genome.

**Figure S13** The types and numbers of SVs in different diploid cotton using the K_2_ reference.

**Figure S14** The types and numbers of SVs in different diploid cotton using the D_5-502_ reference.

**Figure S15** The types and numbers of SVs in different diploid cotton using the A_2_ reference.

**Figure S16** The distributions of SVs in different diploid cotton genomes.

**Figure S17** Investigation of foliar nectary in 17 diverse cotton species.

**Figure S18** The expression level of *GoNe_1_* (**A**) and *GoNe_2_* (**B**) in five diverse cotton species.

**Figure S19** Sequence differences of CDS from *GoNe_1_* (for A subgroup) and *GoNe_2_* (for D subgroup) in the diploid cotton species.

**Figure S20** Sequence differences of promoter from *GoNe_1_* (for A subgroup) and *GoNe_2_* (for D subgroup) in the diploid cotton species.

**Supplemental Tables（TableS.xlsx）**

**Table S1.** Information and assessment of twenty-three genomes.

**Table S2.** The masked sequence and numbers of transposable elements and TE length/assembly length in different cotton species.

**Table S3.** The IDs and information of genes in our pan-genome.

**Table S4.** The gene PAVs in different diploid cottons.

**Table S5.** The gene number in genome numbers of different diploid cotton.

**Table S6.** Pathway and gene ontology (GO) enrichment of core genes.

**Table S7.** The specific gene number to each assembly of diploid cotton.

**Table S8.** Pathway and gene ontology (GO) enrichment of specific genes unique in A_1_ genome.

**Table S9.** Pathway and gene ontology (GO) enrichment of specific genes unique in A_2_ genomes.

**Table S10.** Pathway and gene ontology (GO) enrichment of specific genes unique in D_5_ genomes.

**Table S11.** Pathway and gene ontology (GO) enrichment of specific genes unique in D genomes except D_5_.

**Table S12.** Pathway and gene ontology (GO) enrichment of specific genes unique in other genomes except A and D genomes.

**Table S13.** Pathway and gene ontology (GO) enrichment of specific genes unique in Kirkii genome.

**Table S14.** The size range and number of SVs in different diploid cotton using the K_2_ reference.

**Table S15.** The size range and number of SVs in different diploid cotton using the D_5-502_ reference.

**Table S16.** The size range and number of SVs in different diploid cotton using the A_2_ reference.

**Table S17.** Location of the detected SVs on the genome of 22 cotton species.

**Table S18.** List of 321 SV hotspot regions and 90 genes associated with fiber initiation or/and elongation.

**Table S19.** Oligonucleotides used for qRT-PCR in this study.

**Supplemental References**

**Supplemental Methods**

**Materials and sequencing data**

The assembled genomes of twenty-two diploid cotton and one phylogenetic outgroup species *Gossypioides kirkii* (Kirkii) were downloaded from the CottonGen (<https://www.cottongen.org/>) and the detailed information is included in **Table S1**. BUSCO (v5.4.3) [1] was used to evaluate the assembly completeness of twenty-seven cotton genomes with 1,614 Embryophyta benchmarking universal single-copy orthologs.

Fresh cotton species leaves were collected from the Institute of Cotton Research of CAAS/National Wild Cotton germplasm resources Nursery, Sanya, China/National Crop Germplasm Resources Infrastructure (NCGRI). We selected 17 cotton species representing the seven diploid cotton groups (A, B, D, E, F, G, and K genomes). Detailed information about sample collection is presented in **Table S1**. Leaves of two cotton species, including D_6_ and D_11_, did not have any nectaries on the midribs.

**Repeat sequence annotation**

Transposable elements of the 23 assemblies were annotated using EDTA (v1.3) [2]. These combined the raw predictions of LTRharvest (v1.5.10) [3], LTR_FINDER_parallel (v1.0) [4], LTR_retriever (v2.6) [5], Generic Repeat Finder (v1.0) [6], TIR-Learner (v1.23) [7], MITE-Hunter (v1.0) [8], and HelitronScanner (v1.0) [9] with extra basic and advanced filters. The EDTA-generated nonredundant TE library of each genome was masked using additional databases (lxz.replib.fa) [10]. The unmasked sequences were considered previously unidentified TEs sequences and combined from the 23 TEs libraries. Redundant previously unidentified TEs were removed using the cleanup_nested.pl script in the EDTA package with parameters ‘‘-cov 0.9 -minlen 80 -iter 8.’’ Finally, non-redundant previously unidentified TEs were combined with the TE library to construct the pan-genome TE library [11].

**Phylogenetic tree construction**

Protein sequences of annotated genes from twenty-two genomes [one *G. herbaceum* (A_1_), one *G. arboreum* (A_2_), three *G. raimondii* (D_5_), two *G. thurberi* (D_1_), one for each of *G. armourianum* (D_2-1_), *G. harknessii* (D_2-2_), *G. davidsonii* (D_3d-8_), *G. aridum* (D_4_), *G. gossypioides* (D_6_), *G. lobatum* (D_7_), *G. trilobum* (D_8_), *G. laxum* (D_9_), *G. turneri* (D_10_), *G. schwendimanii* (D_11_), *G. anomalum* (B_1_), *G. stocksii* (E_1_)**,** *G. longicalyx* (F_1_), *G. austral* (G_2_), and *G. rotundifolium* (K_2_)] were analyzed to determine orthology (**Table S1**). The longest proteins for each gene were used in an all-versus-all BLASTP with an E-value cutoff of 1e-5. OrthoFinder (v2.5.2) [12, 13] was used to detect orthogroups of homologous genes from all genomes using default parameters. Single-copy gene orthogroups were aligned with mafft (v7.480) [14] and concatenated into a super alignment. ProtTest (v3.4.2) [15] was used for the maximum likelihood (ML) estimation of phylogenetic trees and model parameters with the parameters: “-all-distributions -F -AIC -BIC -tc 0.5 -threads 24”. RAxML (v8.0.19) [16] was used to build a phylogenetic tree with the command: “raxmlHPC-PTHREADS-SSE3 -T 30 -f a -x 123 -p 123 -N 10000 -m PROTGAMMAJTTF -k -O”. Divergence times were estimated using r8s [17] for orthologous genes using default parameters with the fossil calibration time of *G. herbaceum and G. raimondii* (Median Time: 4.06 MYA, CI: 3.9-4.2 MYA) from the website (<http://www.timetree.org/>).

**Collinearity analysis for 23 assemblies**

To determine the genomic collinearity of gene singletons and pairs in all assemblies, TGT (Triticeae-Gene Tribe9) was used for the 22 assemblies to trace the evolutionary history of the target genes and for gene pairwise collinearity analysis [18]. We used each assembly as a reference for the other assemblies to show the collinearity of all assemblies. In this way, the singleton genes, SBH genes, and RBH genes of each assembly, and the collinearity block between any two assemblies were obtained.

**Ancestor genome construction and synteny block fission and fusions**

The representative genome releases for each diploid species were selected based on the LAI values of each genome assembly. The block adjacencies were produced by transformation of the syntenic block (orthologous conserved segment) sequences with Drimm [19]. To do this, the whole genome CDS of the representative genome were input, using a WGD parameter of 1 for each genome given no lineage-specific WGD among the diploids and Kirkii. The Inferring Ancestor Genome Structure (IAGS) framework [20] was invoked to construct *Gossypium* ancestor genome with the GMP model. The *Gossypium* out group Kirkii was used as guidance for *Gossypium* ancestor genome construction. The CRE (completely rearranged endpoint) ratio of the ancestor genome was 0.75%, with estimated accuracy of 99.76 %.

**Gene-based pan-genome construction**

We employed a stepwise strategy to build the gene-based pan-genome. First, we carried out pairwise collinearity analysis for the 22 assemblies using Triticeae-Gene Tribe9 [18] with default parameters. We used the genes from *G. rotundifolium* (K_2_) as the base, and then the genes from the 22 assemblies were added in a stepwise manner: a gene was added to the gene list and assigned a new locus ID if it was not collinear with any genes in the gene list produced by the preceding step. This operation was repeated until all genes from 22 assemblies were added to the pan-genome.

**KEGG pathway and GO enrichment analysis**

To perform enrichment analysis on the core and specific genes, the core genes present in all 22 assembly genomes and specific genes unique to each assembly of cotton were subjected to KEGG pathway and GO enrichment analysis using the module “Gene-list Enrichment” in KOBAS (<http://kobas.cbi.pku.edu.cn/>) [21] with the default parameters. The significant threshold was corrected *p*-value <= 0.05 using the FDR correction method. The results of pathway and gene ontology (GO) enrichment were plotted using R scripts.

**Identification of SVs**

We aligned the 22 diploid cotton genomes to the *G. rotundifolium* (K_2_), *G. raimondii* (D_5-502_) and *G. arboreum* (A_2_) reference genomes and then identified SVs using the Assemblytics tools (<https://github.com/marianattestad/assemblytics>) [22]. Specifically, the Assemblytics pipeline was follows: 1) obtained alignment pairs from any pair of genomes using nucmer (v3.23) (--maxmatch -c 100 -b 500 -l 50) [22, 23]; 2) filter alignment pairs from any pair of genomes using delta-filter (-m -i 90 -l 100); 3) SVs were identified using Assemblytics with the parameters “10000000 50 10000000”. The types of SVs included “Insertion,” “Deletion,” “Tandem expansion,” “Repeat expansion,” and “Repeat contraction”. We employed a sliding window approach, with a window size of 100 kilobases (kb) and a step size of 50 kb, to quantify the number of structural variations across the entire genome. Subsequently, regions exhibiting structural variation frequencies above 1% were designated as structural variation hotspots. Adjacent hotspots were then merged to consolidate these regions.

**qPCR analysis**

The nectaries on the midribs of leaves from D_5_ and TM-1 and the similar part of the midribs of leaves from D_6_, D_11_, and (AD)_3_ were collected for total RNA extraction using a TIANGEN’s polysaccharides & polyphenolics-rich RNAprep pure plant plus kit. Primers that distinguish the expression between *GoNe_1_* and *GoNe_2_* were designed using WebSNAPER (<http://pga.mgh.harvard.edu/cgi-bin/snap3/websnaper3.cgi>) **(Table S19)**. Reverse transcription for first-strand cDNA synthysis was conducted using the HiScript ⅡQRT SuperMix from Vazyme. ABI 7500 real-time PCR system was used for quantitative real-time polymerase chain reaction (qRT-PCR). The relative transcript levels were calculated using the 2 − ∆∆CT method.

**Data Availability**

The assembled genomes of twenty-seven diploid cotton and one phylogenetic outgroup species *G. kirkii* (Kirkii) were downloaded from the CottonGen (<https://www.cottongen.org/>) and the detailed information (DOI and URL) is included in **Table S1**. The plot code has been submitted to Github (https://github.com/xqwang1990/Cotton_Pangenome_Plot).


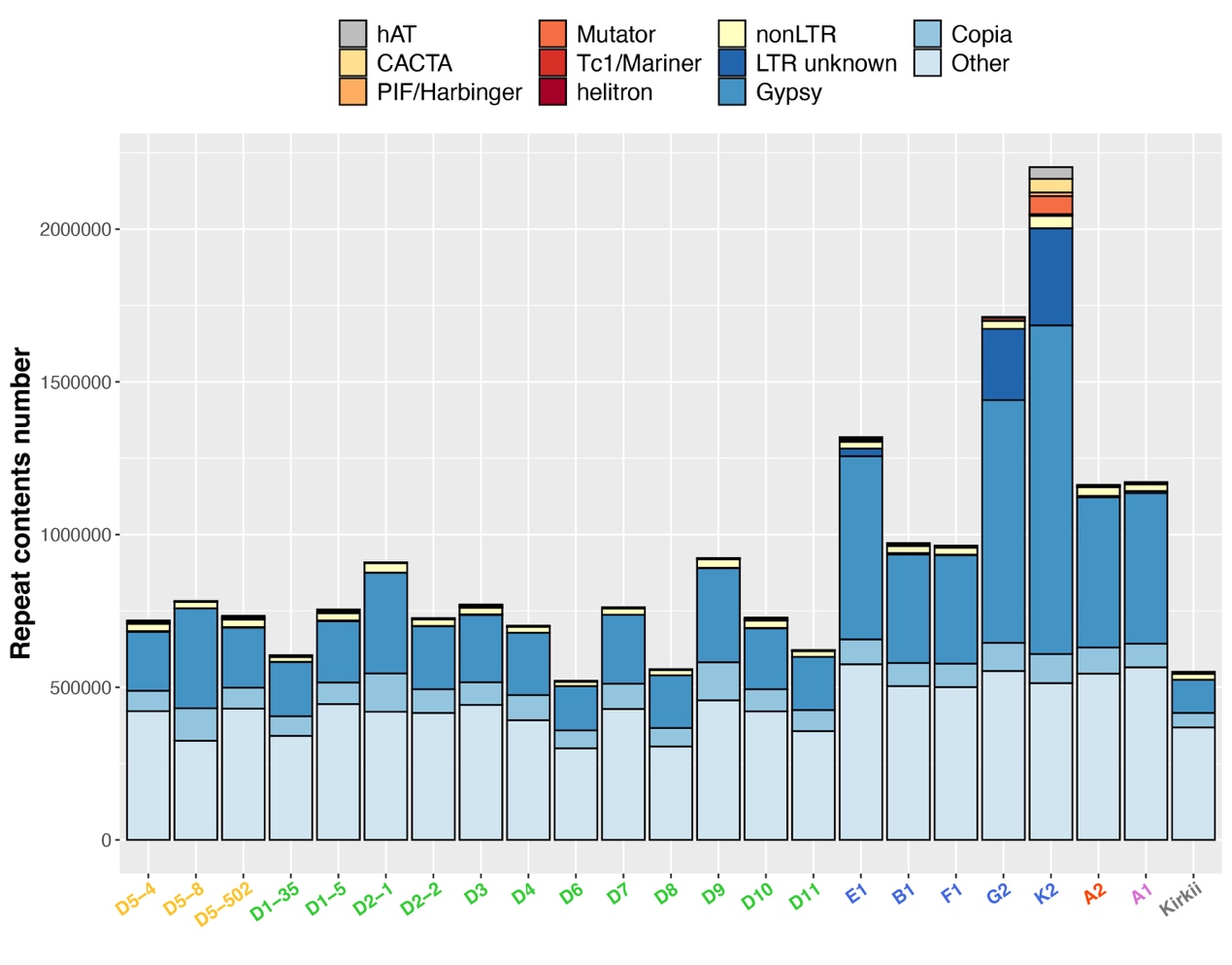


**Figure S1** The types and numbers of transposable elements (TEs) and TE length/assembly length in different cotton species. The different colors of genomes represented different diploid cotton.


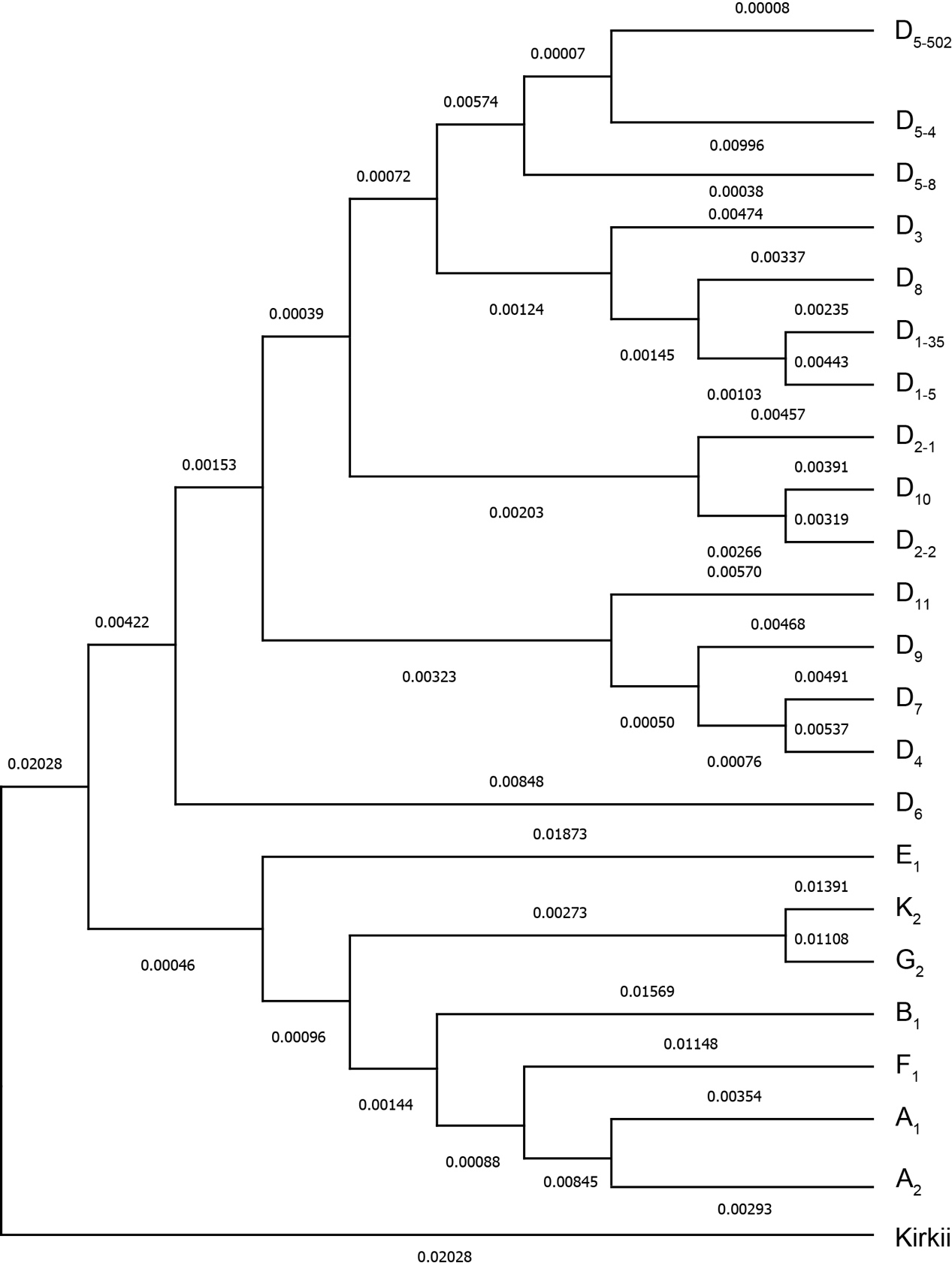


**Figure S2** Phylogenetic tree of twenty-two genomes using 352 single-copy coding genes with the phylogenetic outgroup species *G. kirkii* (Kirkii).

**
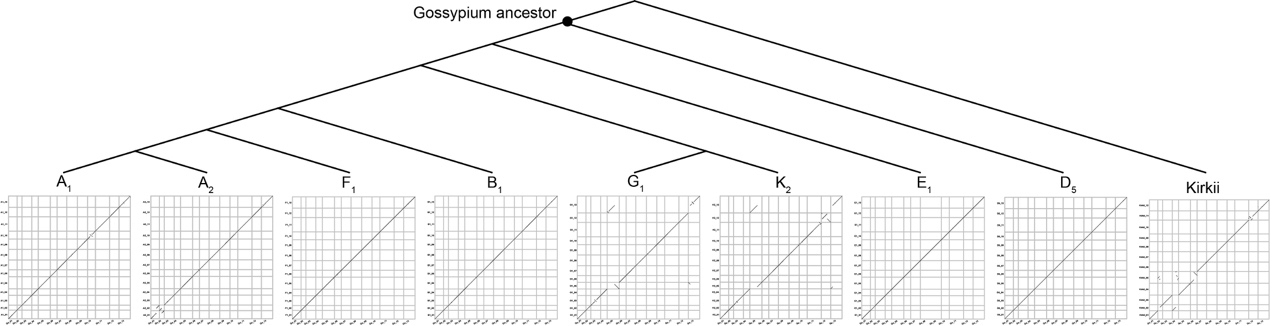
**

**Figure S3** Ancestor genome construction and dot-plot based on the synteny blocks.

**A**


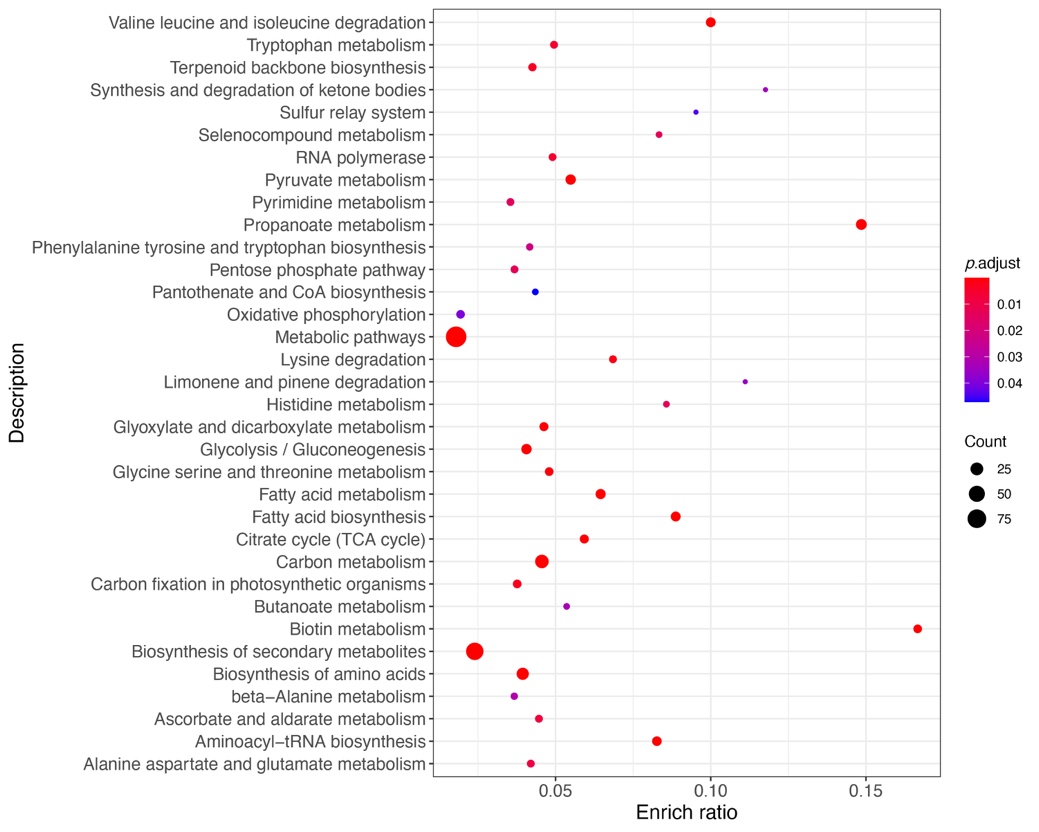


**B**


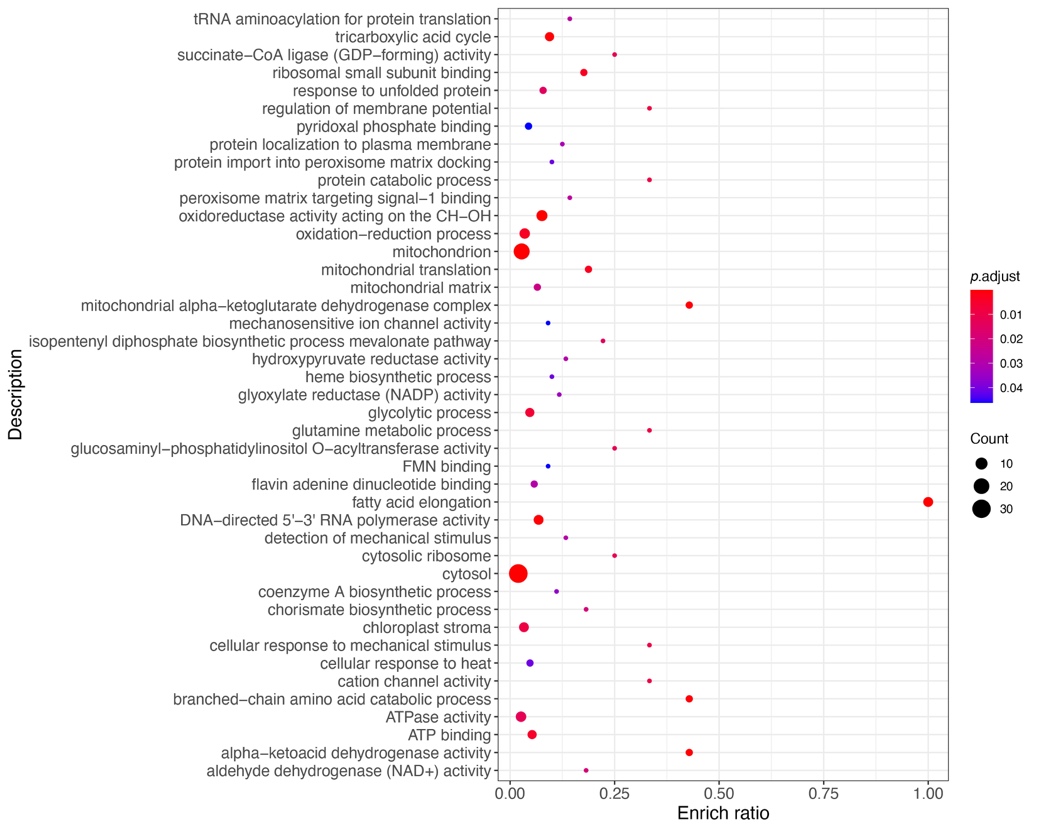


**Figure S4** Pathway **(A)** and Gene ontology (GO) **(B)** enrichment of specific genes unique in A_1_ genome.

**A**


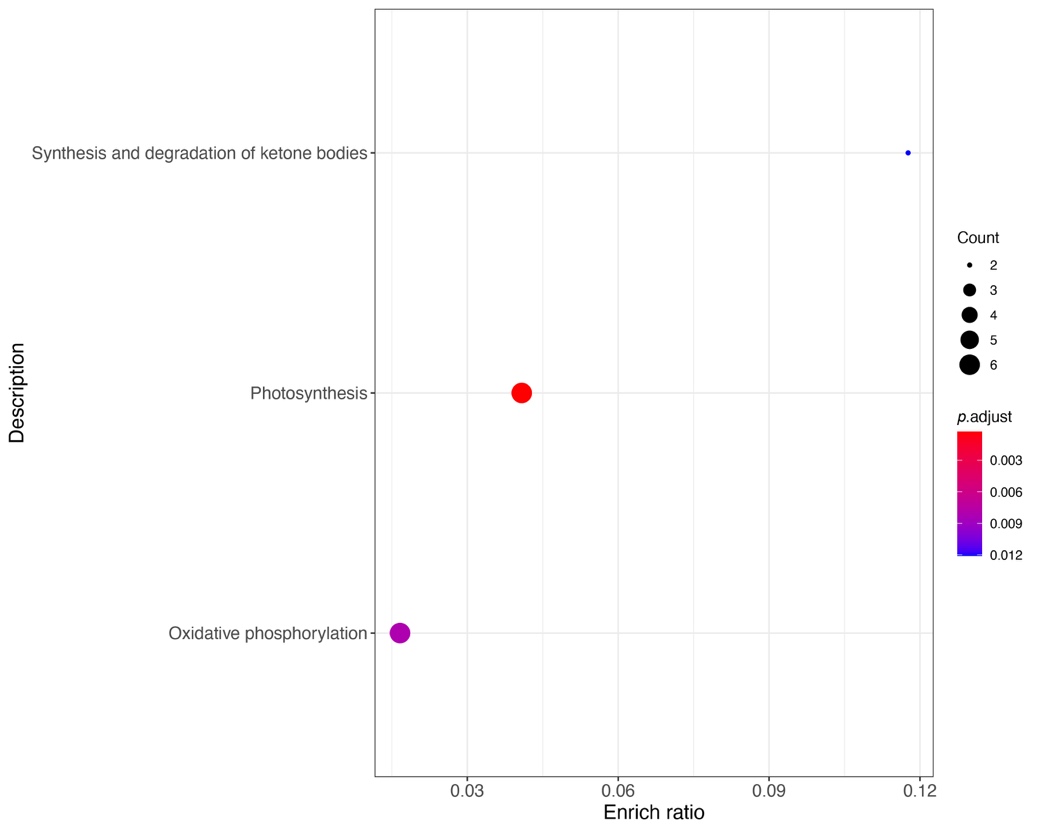


**B**


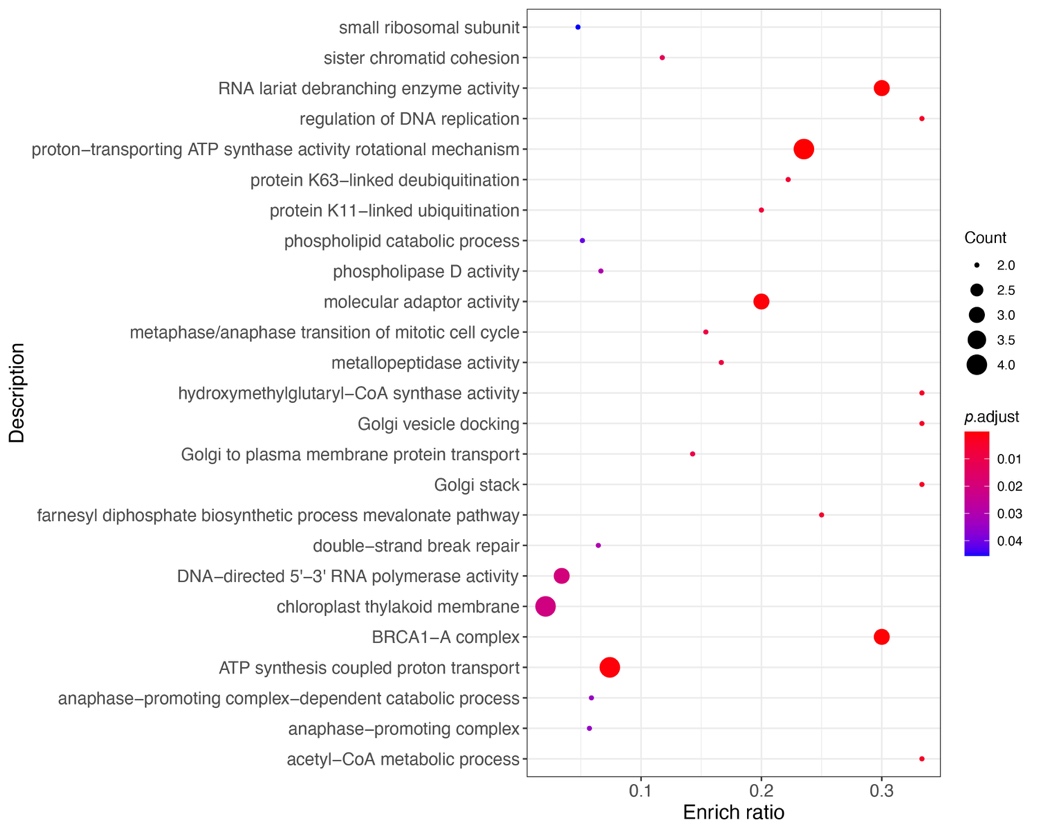


**Figure S5** Pathway **(A)** and Gene ontology (GO) **(B)** enrichment of specific genes unique in A_2_ genomes.


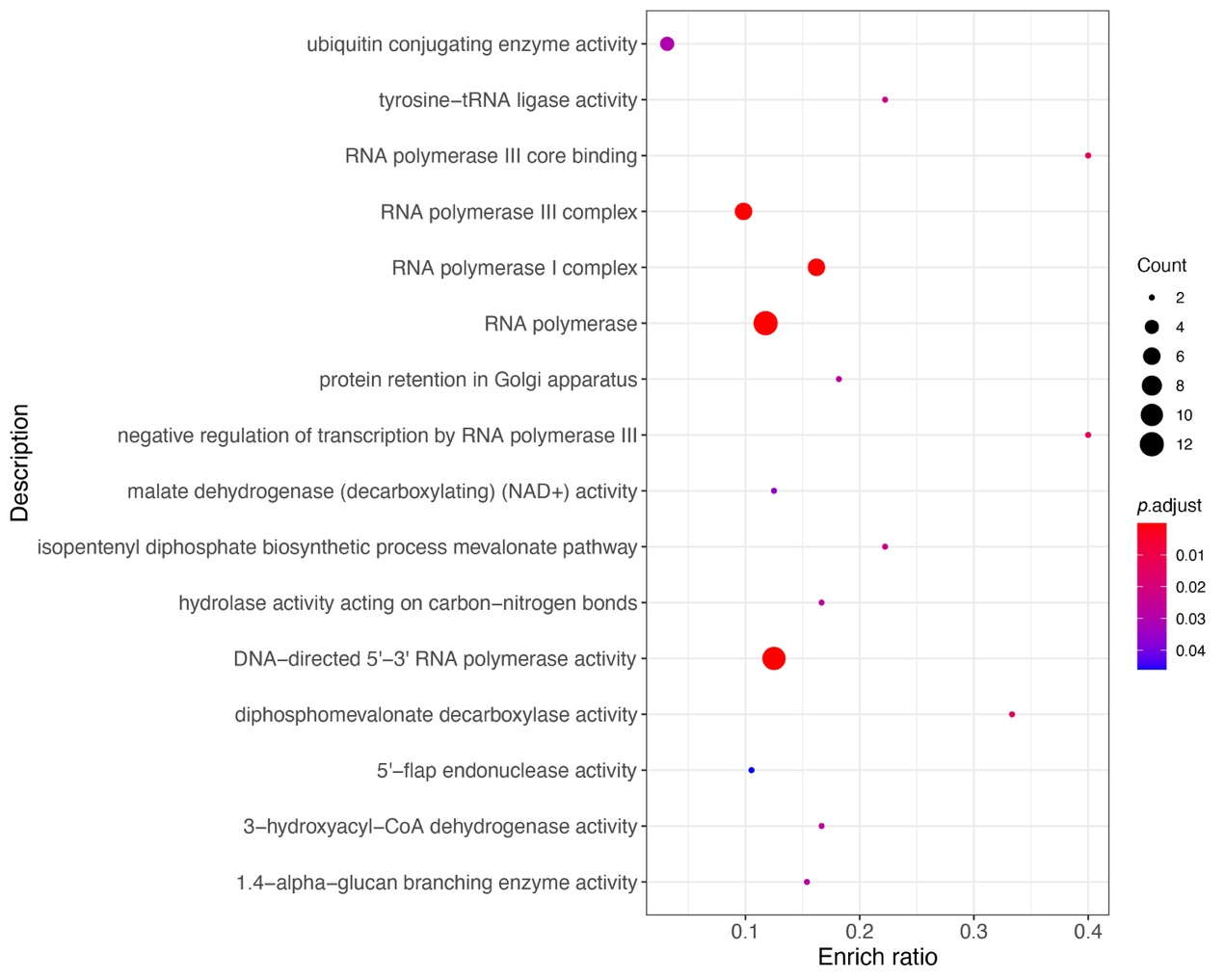


**Figure S6** Pathway and gene ontology (GO) enrichment of specific genes unique in D_5-502_ genome.

**A**

**
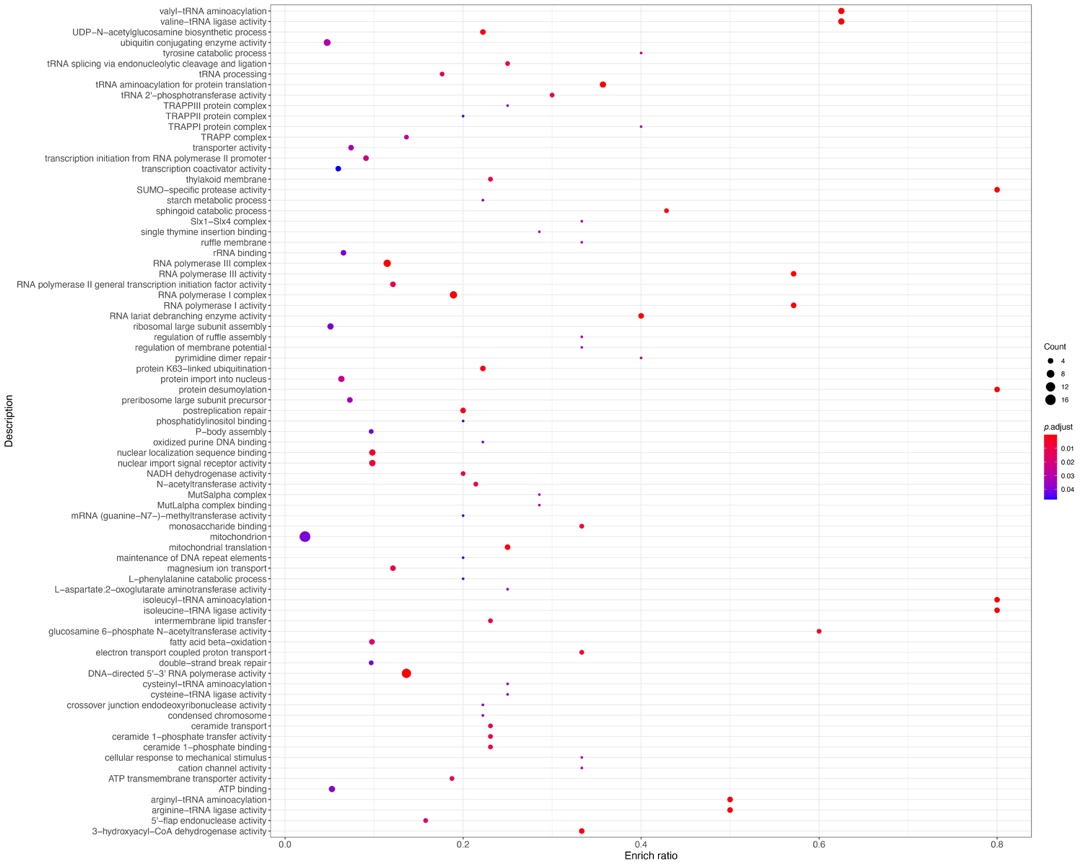
**

**B**


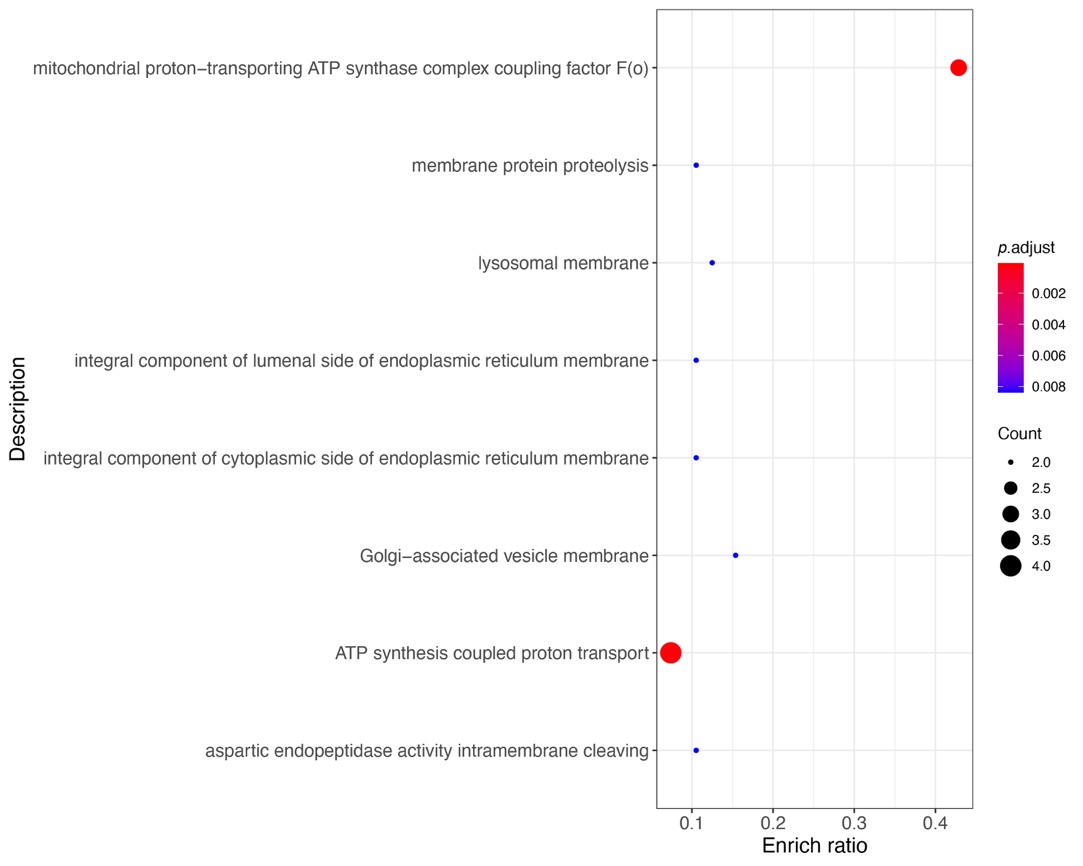


**Figure S7** Gene ontology (GO) enrichment of specific genes unique in D_5-4_ **(A)** and D_5-8_ **(B)** genomes.

**A**

**
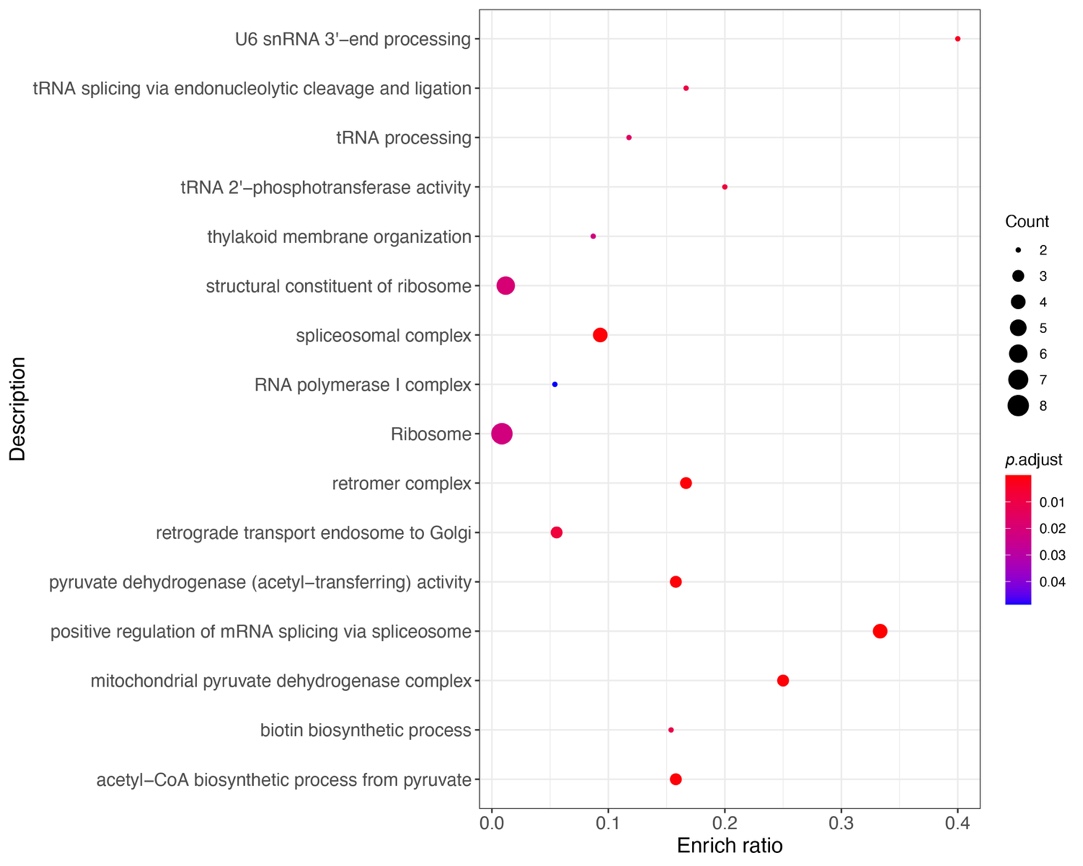
**

**B**


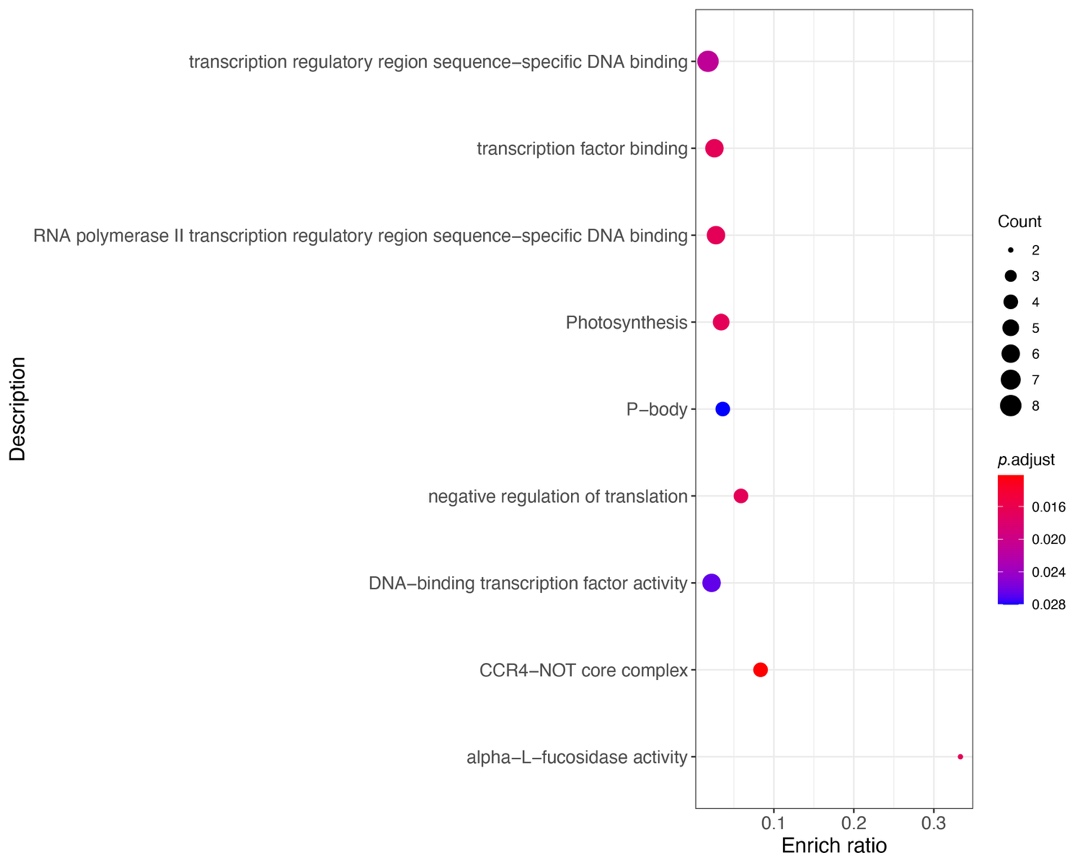


**Figure S8** Pathway and gene ontology (GO) enrichment of specific genes unique in D_1-5_ **(A)** and D_8_ **(B)** genomes.

**A**

**
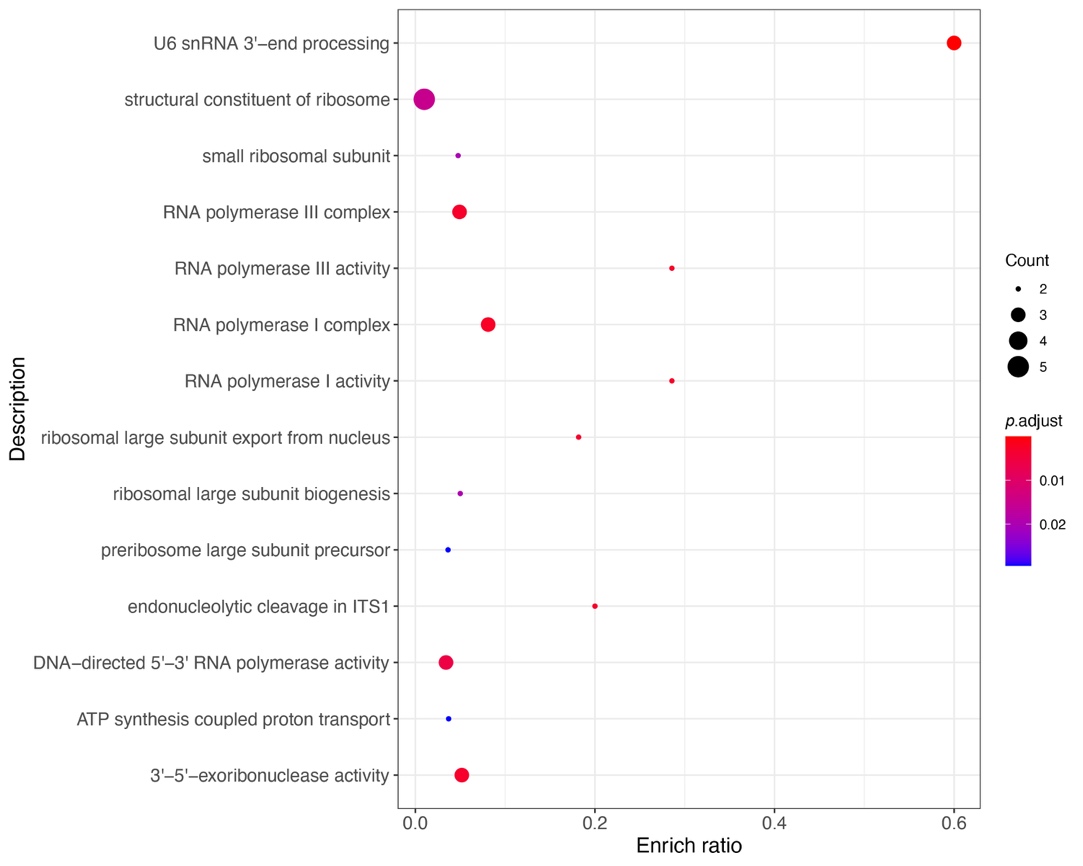
**

**B**


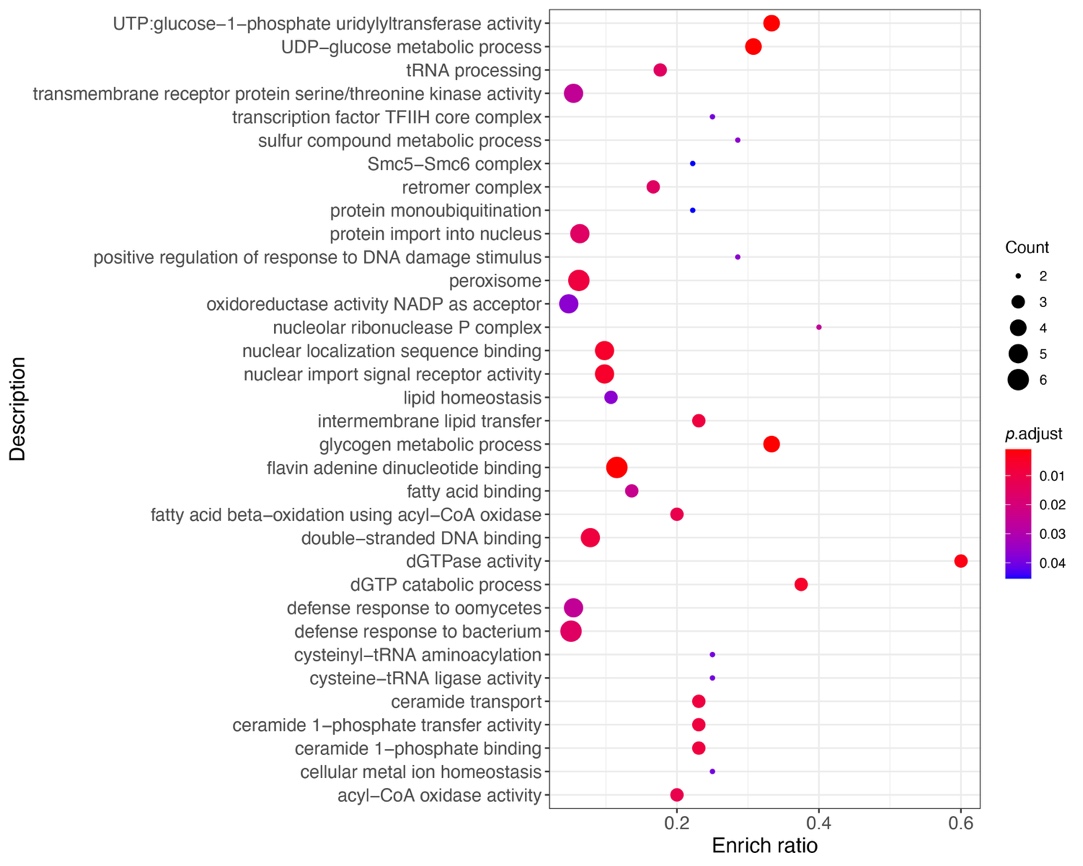


**Figure S9** Gene ontology (GO) enrichment of specific genes unique in D_3_ **(A)** and D_10_ **(B)** genomes.

**A**  **B**


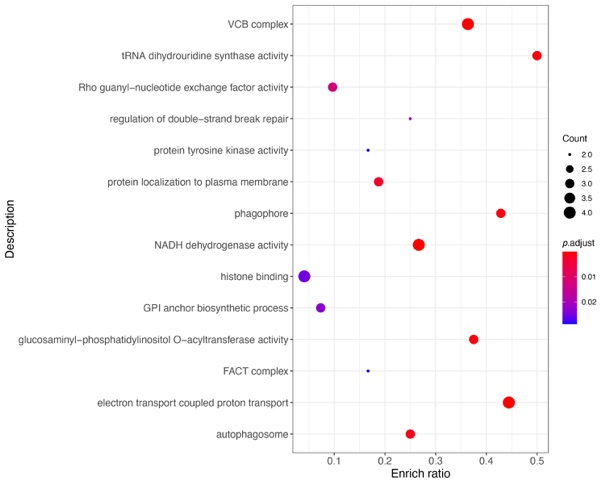

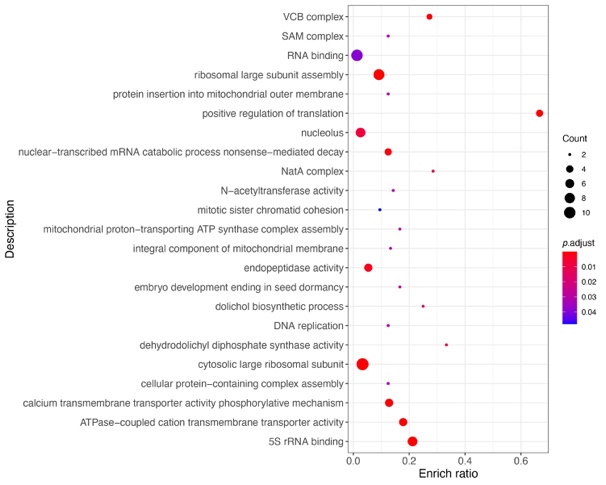


**C**


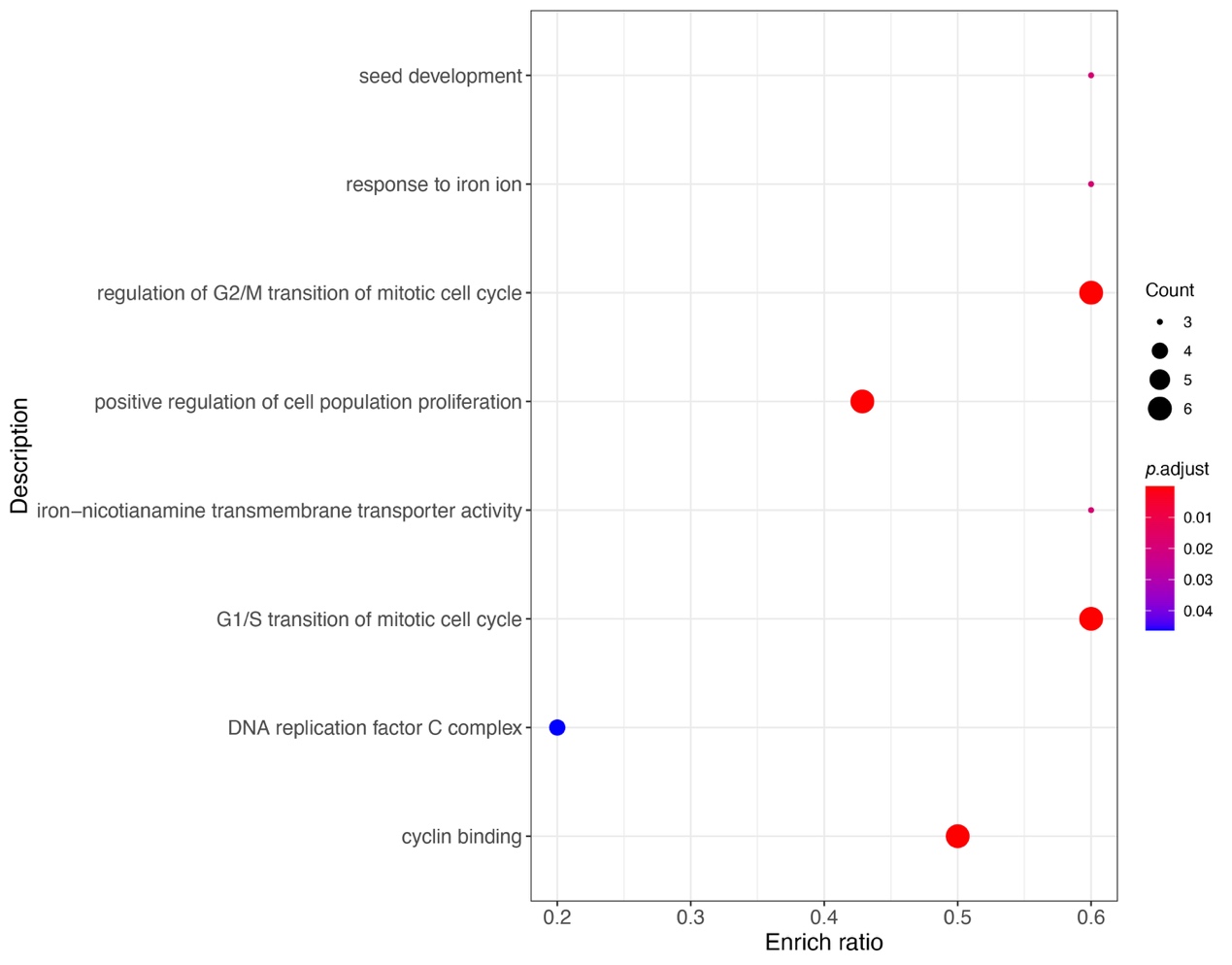


**Figure S101** Gene ontology (GO) enrichment of specific genes unique in B_1_ **(A)**, E_1_ **(B)** and G_2_ **(C)** genomes.

**A B**


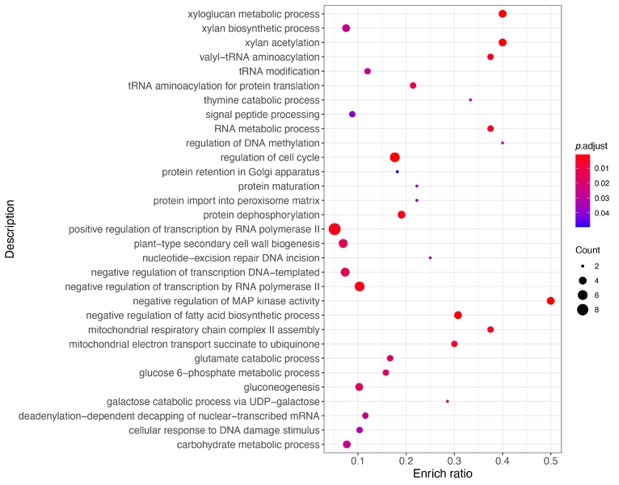

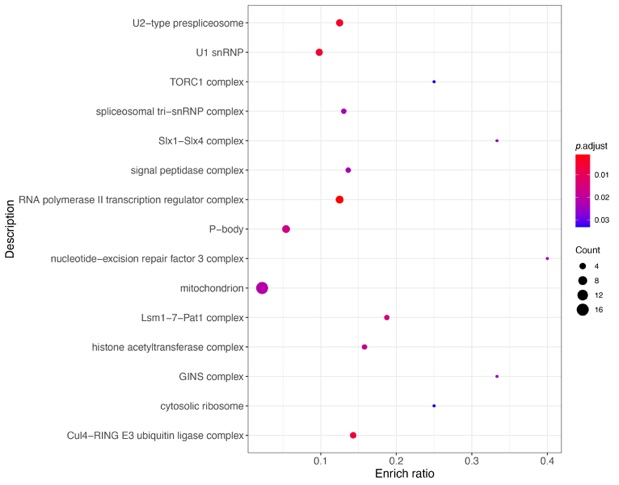


**C**


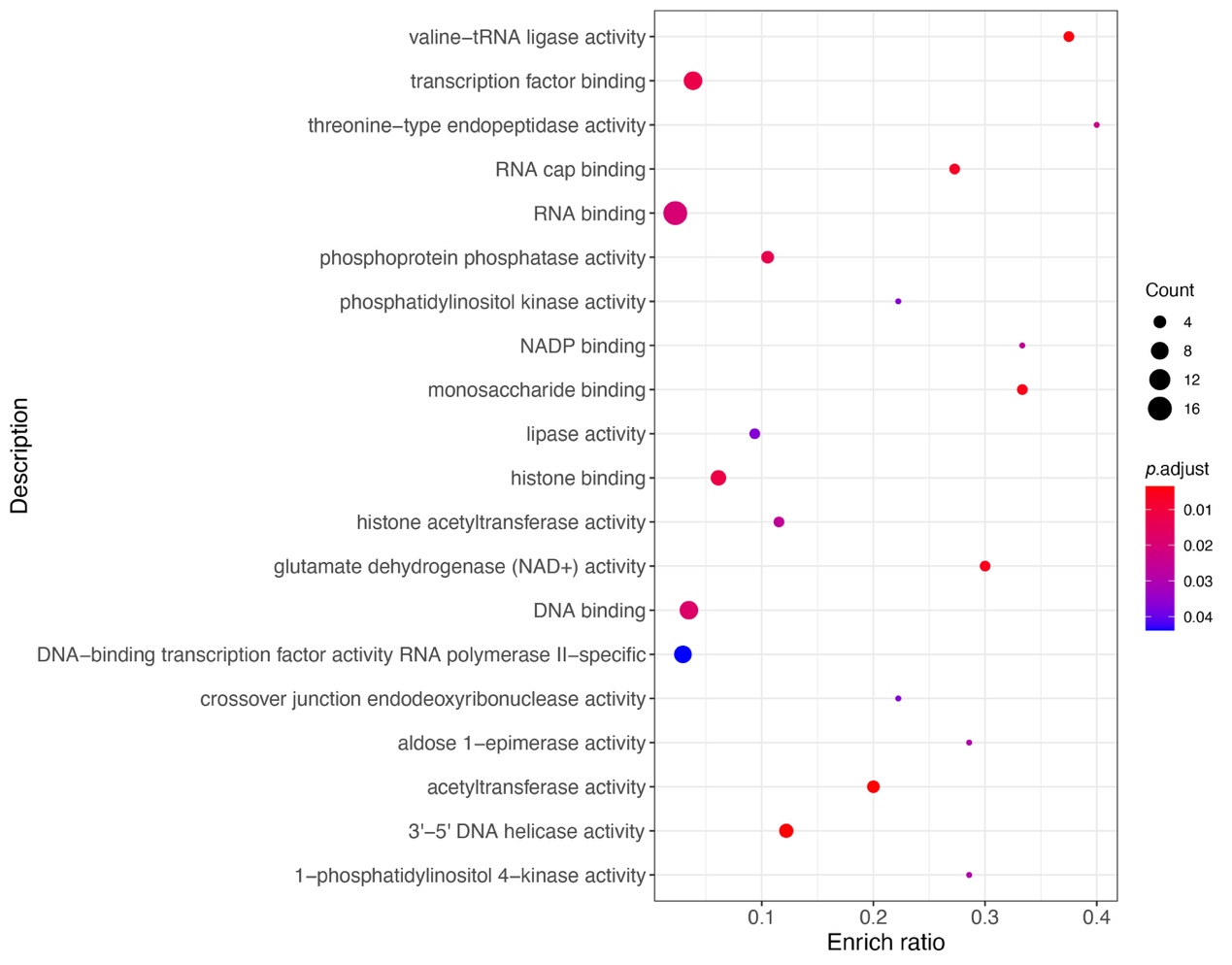


**Figure S11** The biological process **(A)**, cellular component **(B)** and molecular function **(C)** in gene ontology (GO) enrichment of specific genes unique in K_2_ genome.


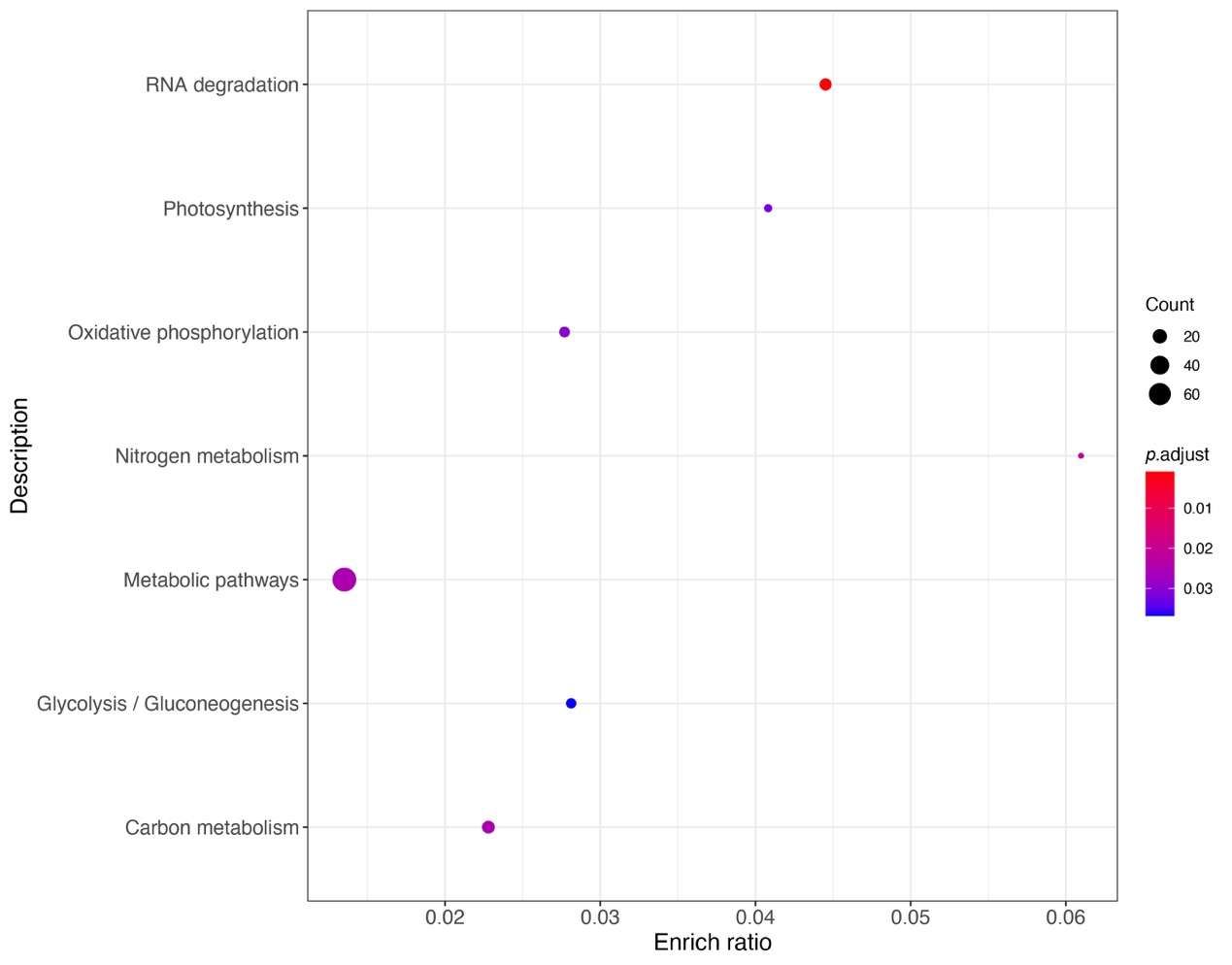


**Figure S12** Pathway enrichment of specific genes unique in K_2_ genome.


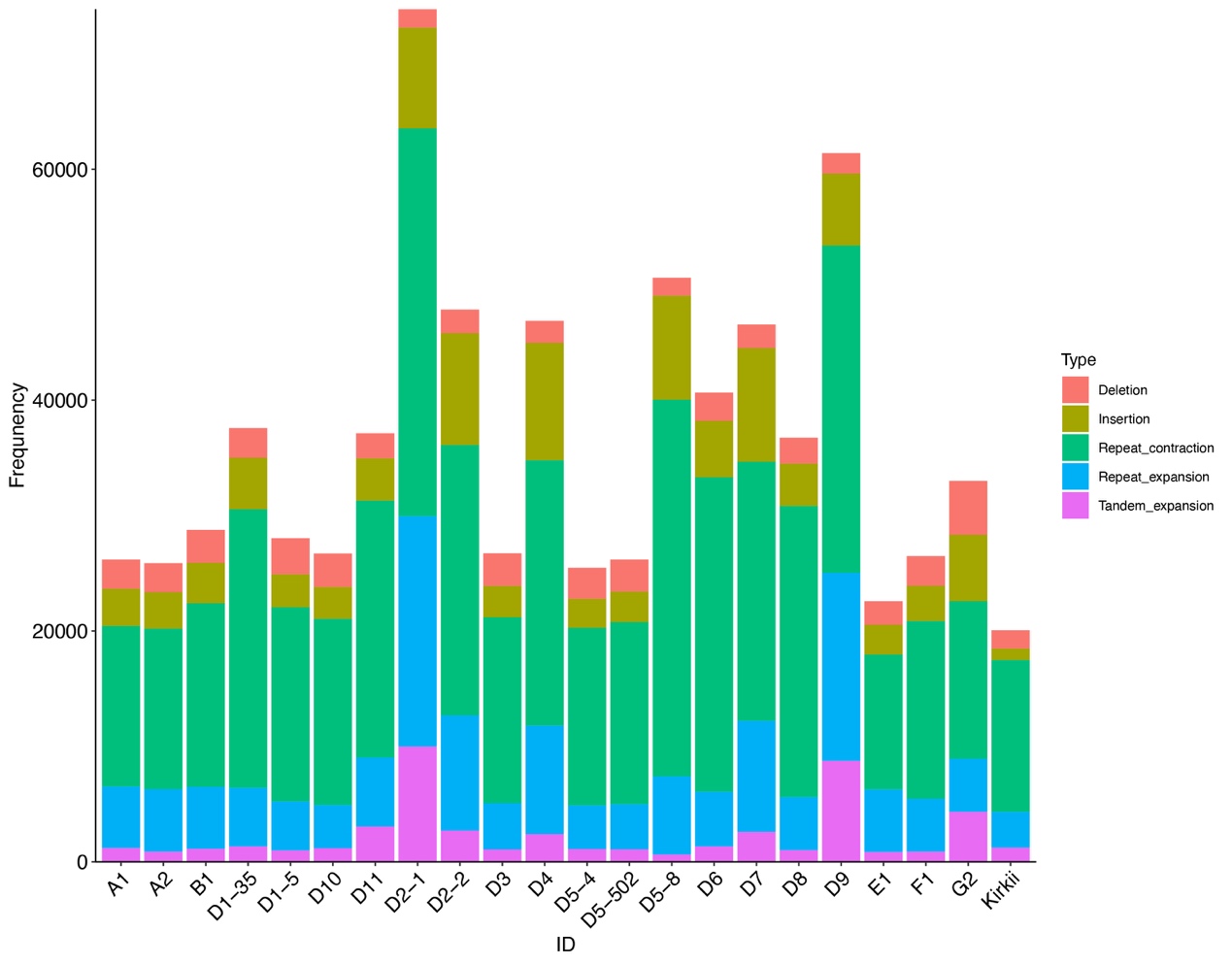


**Figure S13** The types and numbers of SVs in different diploid cotton using the K_2_ reference.


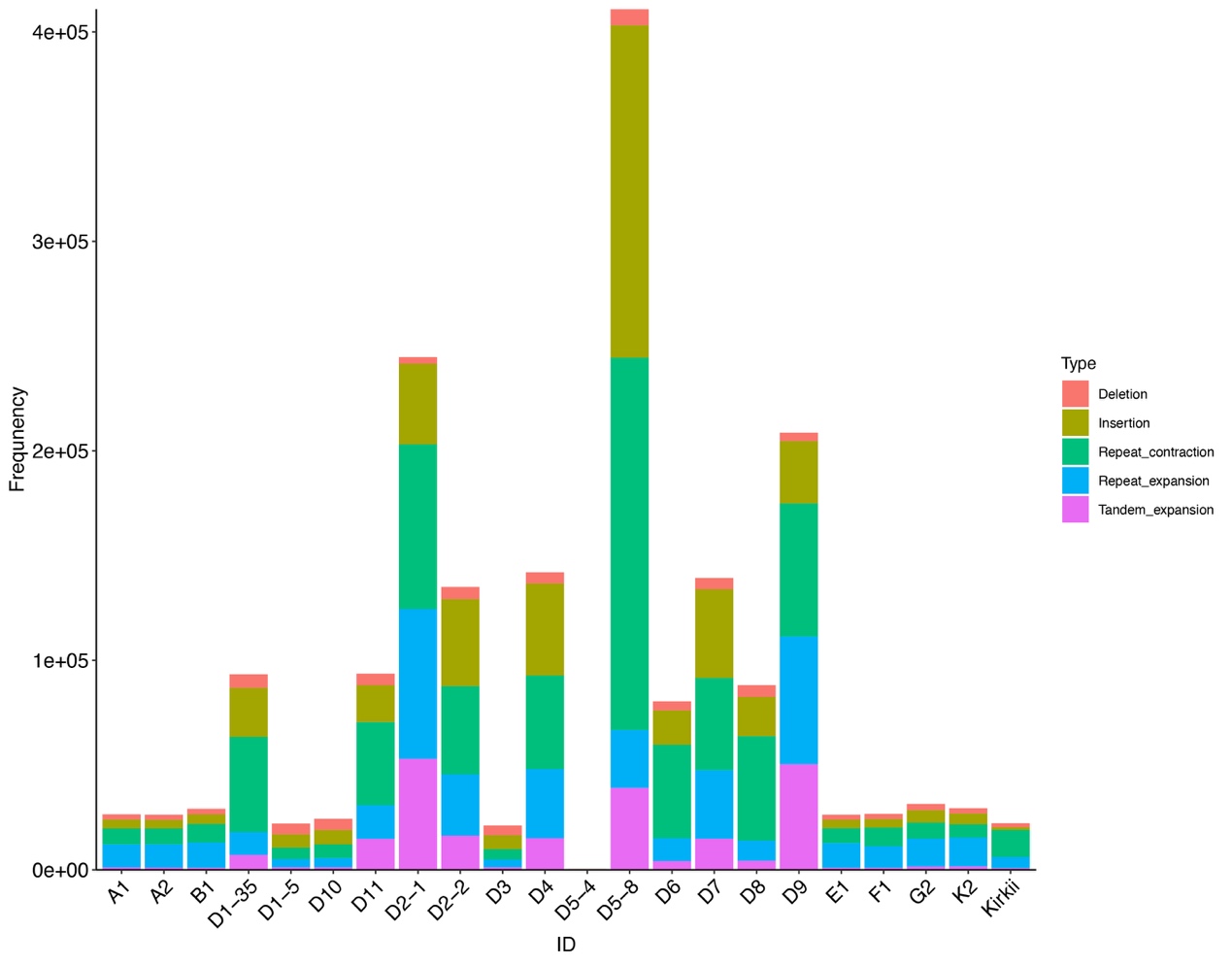


**Figure S14** The types and numbers of SVs in different diploid cotton using the D_5-502_ reference.


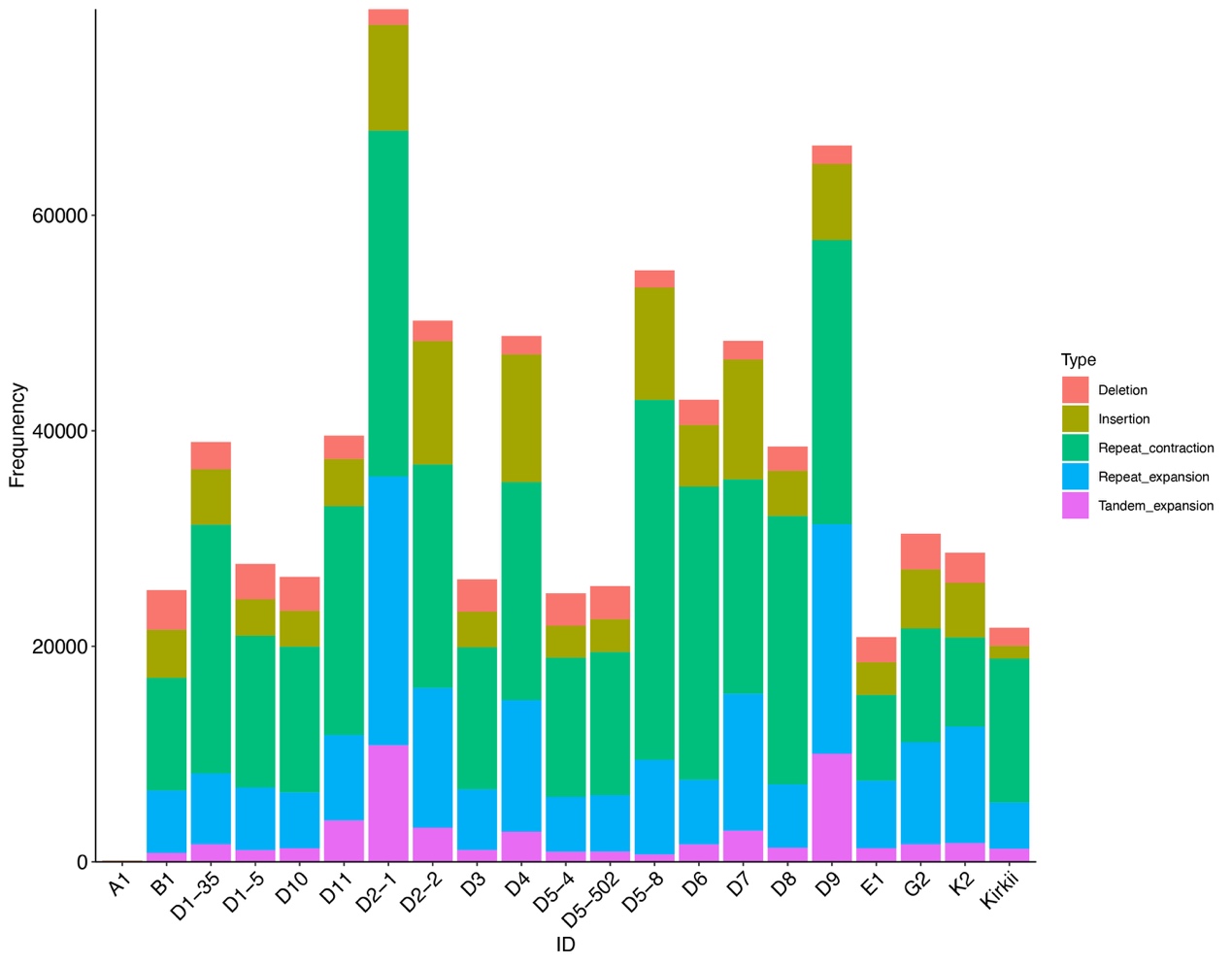


**Figure S15** The types and numbers of SVs in different diploid cotton using the A_2_ reference.

**
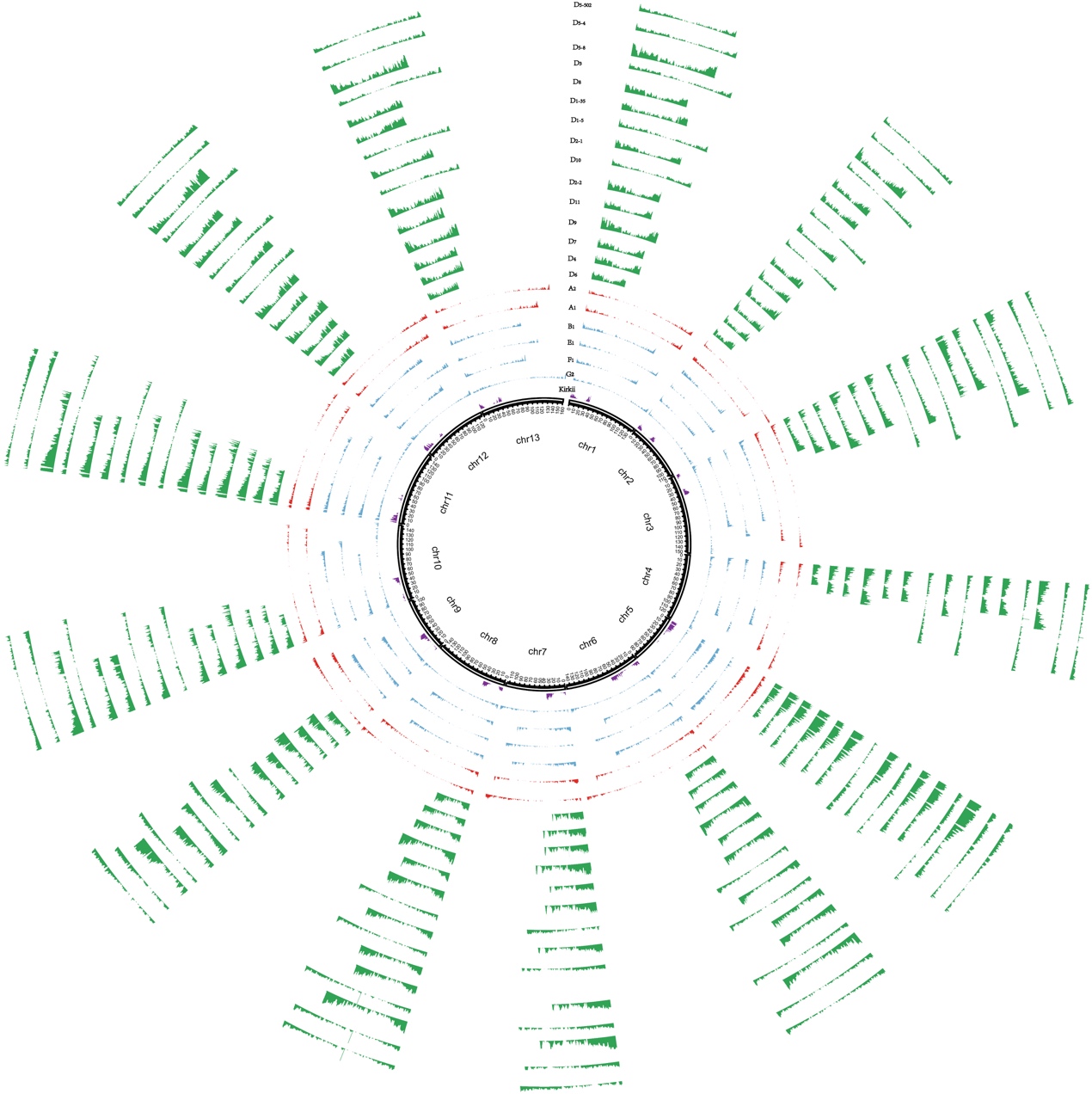
**

**Figure S16** The distributions of SVs in different diploid cotton genomes. The colored histograms in each layer from inner to outer represent respectively the SVs detected from each diploid cotton genome. **Kirkii**: *Gossypioides kirkii*, **G_2_**: *G. austral*, **F_1_**: *G. longicalyx*, **E_1_**: *G. stocksii*, **B_1_**: *G. anomalum*, **A_1_**: *G. herbaceum*, **A_2_**: *G. arboreum*, **D_6_**: *G. gossypioides*, **D_4_**: *G. aridum*, **D_7_**: *G. lobatum*, **D_9_**: *G. laxum*, **D_11_**: *G. schwendimanii*, **D_2-2_**: *G. harknessii*, **D_10_**: *G. turneri*, **D_2-1_**: *armourianum*, **D_1-5_**: *G. thurberi*, **D_1-35_**: *G. thurberi*, **D_8_**: *G. trilobum*, **D_3_**: *G. davidsonii*, **D_5-8_**: *G. raimondii*, **D_5-4_**: *G. raimondii*, **D_5-502_**: *G. raimondii*. Purple represented the outgroup, light blue represented other cotton groups (B, E, F and G genomes), red represented cotton group (A genome), and green represented cotton group (D genome).

**Figure S17** Investigation of foliar nectary in 17 diverse cotton species. **A** From the left to the right, it's A_1_, A_2_, D_1_, D_2-1_, D_3_; **B** From the left to the right, it's D_4_, D_5_, D_6_, D_7_, D_8_; **C** From the left to the right, it's D_9_, D_11_, B_1_, E_1_, F_1_; **D** From the left to the right, it's G_2_, K_2_. The red frame represented foliar nectary in each species. D_6_ and D_11_ did not have any nectaries on the midribs of leaves. The scale bar represented 2 cm.


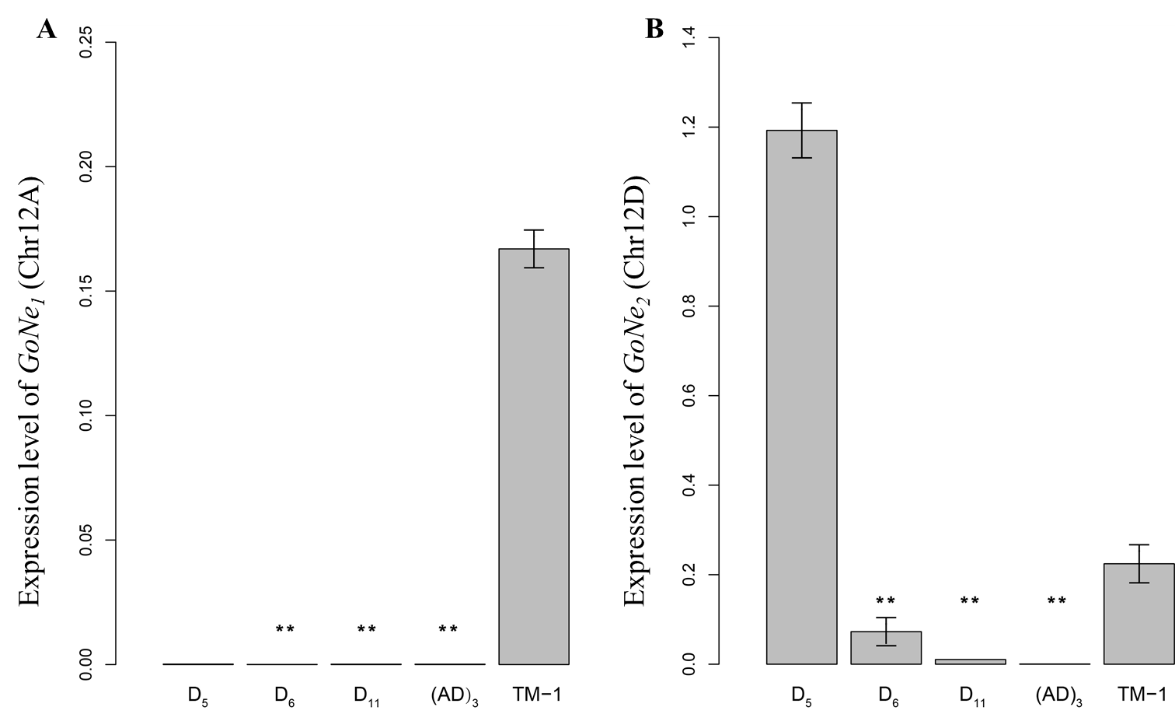


**Figure S18** The expression level of *GoNe_1_* (**A**) and *GoNe_2_* (**B**) in five diverse cotton species.

** indicate significant differences (*p* < 0.05) detected by one-way ANOVA between have foliar nectary (TM-1 and D_5_) to no foliar nectary ((AD)_3_, D_6_ and D_11_).


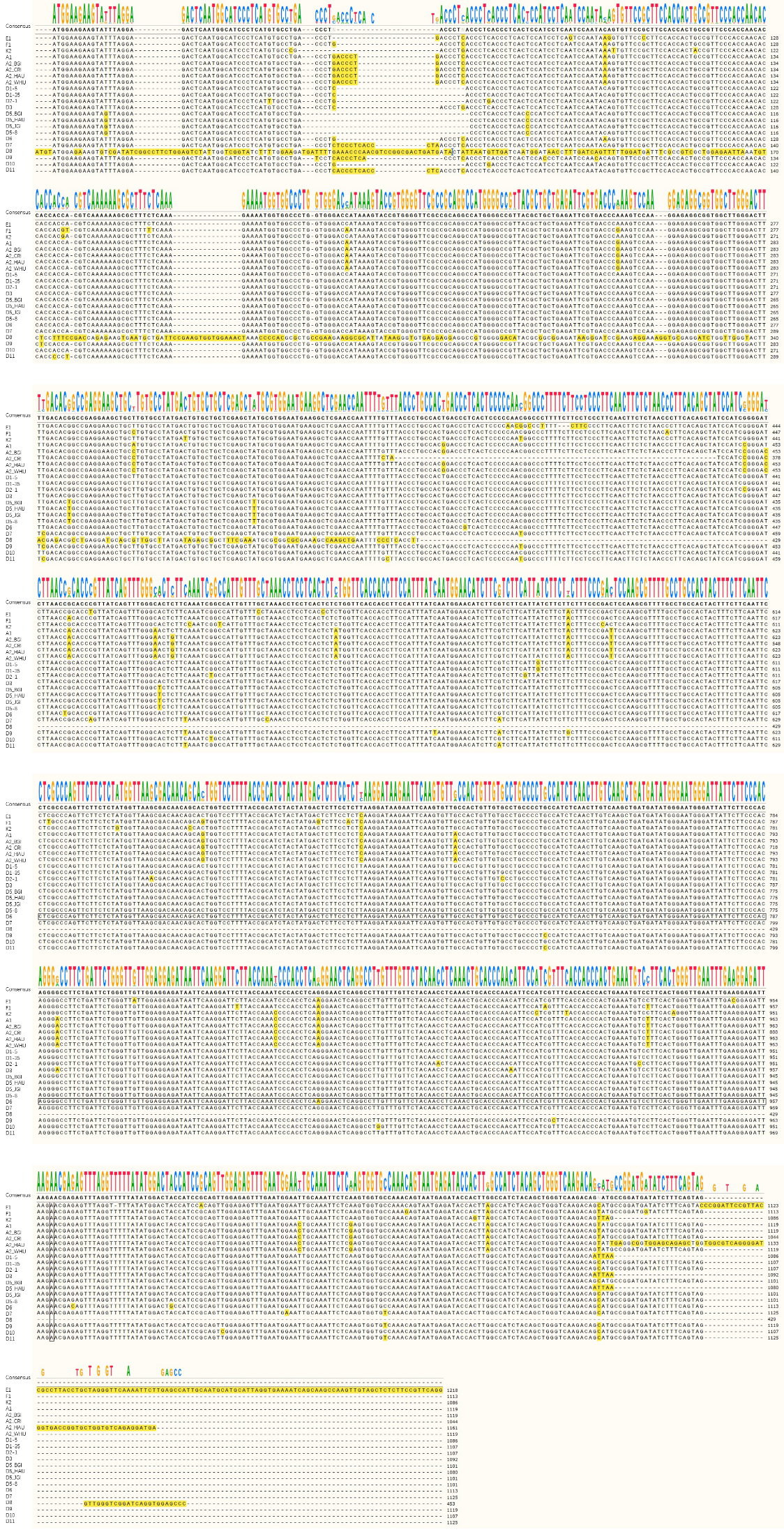


**Figure S19** Sequence differences of CDS from *GoNe_1_* (for A subgroup) and *GoNe_2_* (for D subgroup) in the diploid cotton species.

**
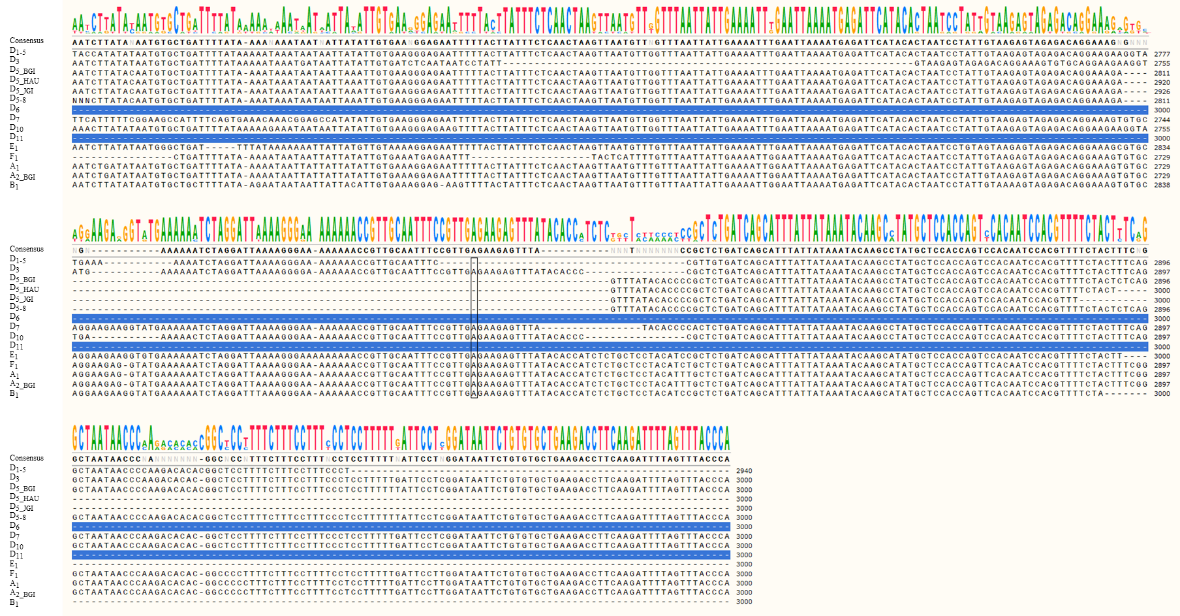
**

**Figure S20** Sequence differences of promoter from *GoNe_1_* (for A subgroup) and *GoNe_2_* (for D subgroup) in the diploid cotton species.

**Supplemental References**

1. Manni, Mosè, Matthew R Berkeley, Mathieu Seppey, Felipe A Simão, Evgeny M Zdobnov. 2021. “BUSCO Update: Novel and Streamlined Workflows along with Broader and Deeper Phylogenetic Coverage for Scoring of Eukaryotic, Prokaryotic, and Viral Genomes.” *Molecular Biology And Evolution* 38: 4647-4654. <https://doi.org/10.1093/molbev/msab199>

2. Ou, Shujun, Weija Su, Yi Liao, Kapeel Chougule, Jireh R. A. Agda, Adam J. Hellinga, Carlos Santiago Blanco Lugo, et al. 2019. “Benchmarking transposable element annotation methods for creation of a streamlined, comprehensive pipeline.” *Genome Biology* 20: 275. <https://doi.org/10.1186/s13059-019-1905-y>

3. Ellinghaus, David, Stefan Kurtz, Ute Willhoeft. 2008. “LTRharvest, an efficient and flexible software for de novo detection of LTR retrotransposons.” *BMC Bioinformatics* 9: 18. <https://doi.org/10.1186/1471-2105-9-18>

4. Ou, Shujun, Ning Jiang. 2019. “LTR_FINDER_parallel: parallelization of LTR_FINDER enabling rapid identification of long terminal repeat retrotransposons.” *Mobile DNA* 10: 48. <https://doi.org/10.1186/s13100-019-0193-0>

5. Ou, Shujun, Ning Jiang. 2017. “LTR_retriever: A Highly Accurate and Sensitive Program for Identification of Long Terminal Repeat Retrotransposons.” *Plant Physiology* 176: 1410-1422. <https://doi.org/10.1104/pp.17.01310>

6. Shi, Jieming, Chun Liang. 2019. “Generic Repeat Finder: A High-Sensitivity Tool for Genome-Wide De Novo Repeat Detection.” *Plant Physiology* 180: 1803-1815. <https://doi.org/10.1104/pp.19.00386>

7. Su, Weijia, Xun Gu, Thomas Peterson. 2019. “TIR-Learner, a New Ensemble Method for TIR Transposable Element Annotation, Provides Evidence for Abundant New Transposable Elements in the Maize Genome.” *Molecular Plant* 12: 447-460. <https://doi.org/10.1016/j.molp.2019.02.008>

8. Han, Yujun, Susan R. Wessler. 2010. “MITE-Hunter: a program for discovering miniature inverted-repeat transposable elements from genomic sequences.” *Nucleic Acids Research* 38: e199-e199. <https://doi.org/10.1093/nar/gkq862>

9. Xiong, Wenwei, Limei He, Jinsheng Lai, Hugo K. Dooner, Chunguang Du. 2014. “HelitronScanner uncovers a large overlooked cache of <i>Helitron</i> transposons in many plant genomes.” *Proceedings of the National Academy of Sciences* 111: 10263-10268. <https://doi.org/doi:10.1073/pnas.1410068111>

10. Luo, Xizhi, Shiyu Chen, Yu Zhang. 2022. “PlantRep: a database of plant repetitive elements.” *Plant Cell Reports* 41: 1163-1166. <https://doi.org/10.1007/s00299-021-02817-y>

11. Hufford, Matthew B., Arun S. Seetharam, Margaret R. Woodhouse, Kapeel M. Chougule, Shujun Ou, Jianing Liu, William A. Ricci, et al. 2021. “De novo assembly, annotation, and comparative analysis of 26 diverse maize genomes.” *Science* 373: 655-662. <https://doi.org/10.1126/science.abg5289>

12. Emms, David M., Steven Kelly. 2019. “OrthoFinder: phylogenetic orthology inference for comparative genomics.” *Genome Biology* 20: 238. <https://doi.org/10.1186/s13059-019-1832-y>

13. Emms, David M., Steven Kelly. 2015. “OrthoFinder: solving fundamental biases in whole genome comparisons dramatically improves orthogroup inference accuracy.” *Genome Biology* 16: 157. <https://doi.org/10.1186/s13059-015-0721-2>

14. Katoh, Kazutaka, Daron M. Standley. 2013. “MAFFT Multiple Sequence Alignment Software Version 7: Improvements in Performance and Usability.” *Molecular Biology And Evolution* 30: 772-780. <https://doi.org/10.1093/molbev/mst010>

15. Darriba, Diego, Guillermo L. Taboada, Ramón Doallo, David Posada. 2011. “ProtTest 3: fast selection of best-fit models of protein evolution.” *Bioinformatics* 27: 1164-1165. <https://doi.org/10.1093/bioinformatics/btr088>

16. Stamatakis, Alexandros. 2014. “RAxML version 8: a tool for phylogenetic analysis and post-analysis of large phylogenies.” *Bioinformatics* 30: 1312-1313. <https://doi.org/10.1093/bioinformatics/btu033>

17. Sanderson, Michael J. 2003. “r8s: inferring absolute rates of molecular evolution and divergence times in the absence of a molecular clock.” *Bioinformatics* 19: 301-302. <https://doi.org/10.1093/bioinformatics/19.2.301>

18. Chen, Yongming, Wanjun Song, Xiaoming Xie, Zihao Wang, Panfeng Guan, Huiru Peng, Yuannian Jiao, Zhongfu Ni, Qixin Sun, Weilong Guo. 2020. “A Collinearity-Incorporating Homology Inference Strategy for Connecting Emerging Assemblies in the Triticeae Tribe as a Pilot Practice in the Plant Pangenomic Era.” *Molecular Plant* 13: 1694-1708. <https://doi.org/10.1016/j.molp.2020.09.019>

19. Pham, Son K., Pavel A. Pevzner. 2010. “DRIMM-Synteny: decomposing genomes into evolutionary conserved segments.” *Bioinformatics* 26: 2509-2516. <https://doi.org/10.1093/bioinformatics/btq465>

20. Gao, Shenghan, Xiaofei Yang, Jianyong Sun, Xixi Zhao, Bo Wang, Kai Ye. 2022. “IAGS: Inferring Ancestor Genome Structure under a Wide Range of Evolutionary Scenarios.” *Molecular Biology And Evolution* 39: <https://doi.org/10.1093/molbev/msac041>

21. Bu, Dechao, Haitao Luo, Peipei Huo, Zhihao Wang, Shan Zhang, Zihao He, Yang Wu, et al. 2021. “KOBAS-i: intelligent prioritization and exploratory visualization of biological functions for gene enrichment analysis.” *Nucleic Acids Research* 49: W317-W325. <https://doi.org/10.1093/nar/gkab447>

22. Nattestad, Maria, Michael C. Schatz. 2016. “Assemblytics: a web analytics tool for the detection of variants from an assembly.” *Bioinformatics* 32: 3021-3023. <https://doi.org/10.1093/bioinformatics/btw369>

23. Tang, Dié, Yuxin Jia, Jinzhe Zhang, Hongbo Li, Lin Cheng, Pei Wang, Zhigui Bao, et al. 2022. “Genome evolution and diversity of wild and cultivated potatoes.” *Nature* 606: 535-541. <https://doi.org/10.1038/s41586-022-04822-x>
